# Genomes-based phylogeny of the genus *Xanthomonas*

**DOI:** 10.1186/1471-2180-12-43

**Published:** 2012-03-23

**Authors:** Luis M Rodriguez-R, Alejandro Grajales, Mario L Arrieta-Ortiz, Camilo Salazar, Silvia Restrepo, Adriana Bernal

**Affiliations:** 1 Laboratory of Mycology and Plant Pathology, Biological Sciences Department, Universidad de Los Andes, Cra 1 No 18A-12, Bogotá, Colombia; 2 Division of Invertebrate Zoology, American Museum of Natural History, Central Park West at 79th Street, 10024-5192 New York, NY, USA; 3 Smithsonian Tropical Research Institute, Roosvelt Ave. Tupper Building, Panama 0843-03092, Panama; 4 University of Cambridge, The Old Schools, Trinity Lane, Cambridge CB2 1TN, UK; 5 Department of Biology, Georgia Institute of Technology, 311 Ferst Drive, 30332 Atlanta, GA, USA

## Abstract

**Background:**

The genus *Xanthomonas *comprises several plant pathogenic bacteria affecting a wide range of hosts. Despite the economic, industrial and biological importance of *Xanthomonas*, the classification and phylogenetic relationships within the genus are still under active debate. Some of the relationships between pathovars and species have not been thoroughly clarified, with old pathovars becoming new species. A change in the genus name has been recently suggested for *Xanthomonas albilineans*, an early branching species currently located in this genus, but a thorough phylogenomic reconstruction would aid in solving these and other discrepancies in this genus.

**Results:**

Here we report the results of the genome-wide analysis of DNA sequences from 989 orthologous groups from 17 *Xanthomonas *spp. genomes available to date, representing all major lineages within the genus. The phylogenetic and computational analyses used in this study have been automated in a Perl package designated Unus, which provides a framework for phylogenomic analyses which can be applied to other datasets at the genomic level. Unus can also be easily incorporated into other phylogenomic pipelines.

**Conclusions:**

Our phylogeny agrees with previous phylogenetic topologies on the genus, but revealed that the genomes of *Xanthomonas citri *and *Xanthomonas fuscans *belong to the same species, and that of *Xanthomonas albilineans *is basal to the joint clade of *Xanthomonas *and *Xylella fastidiosa*. Genome reduction was identified in the species *Xanthomonas vasicola *in addition to the previously identified reduction in *Xanthomonas albilineans*. Lateral gene transfer was also observed in two gene clusters.

## Background

*Xanthomonas *is a genus in the gamma division of Proteobacteria primarily constituted by pathogens to plants of considerable economic importance. These pathogens affect a wide variety of crops, including *Citrus *spp. (lime, orange, lemon and pomelo, among others), *Oryza *spp. (rice), crucifers (cabbage, broccoli, cauliflower, radish and *Arabidopsis thaliana*) and *Manihot esculenta *(cassava), with individual members showing a high degree of host specificity [1]. *Xanthomonas *is among the few bacterial genera in which large DNA-DNA hybridization, RFLP and REP-PCR datasets are available [2-6] and have been employed for the taxonomical resolution of the group [7]. In addition, the availability of more than ten genomes within the genus [8,9] has allowed recent studies of comparative genomics and genome evolution [10,11].

The genus *Xanthomonas *has been subject to numerous taxonomical and phylogenetic studies, starting with the description of *Bacterium vesicatorium *as the causal agent of bacterial spot on pepper and tomato [12] and its reclassification as *Xanthomonas campestris *[13,14]. *Xanthomonas *was first described as a monotypic genus, and later divided in two groups, A and B [15,16]. A subsequent study [6] classified 183 reported strains into 20 different species mainly based on DNA-DNA hybridization data. Since then, a general classification has been established based on polyphasic analysis [6,17], while other analyses helped to clarify the classification in specific clades, mainly using Multi Locus Sequence Analysis (MLSA) and Amplified Fragment Length Polymorphism (AFLP) [18,19]. This allowed the development of several typing and characterization tools (*e.g*., [11,18-24]), which have revealed the diversity and complexity of the genus [23,24], while showing the limitations of single locus analyses [25]. However, during the last decade the taxonomy of this genus has still been subject to considerable debate. Genus-wide reclassifications have been proposed [26,27], and frequent sub-specific reclassifications and proposals for new species have been published [19-21,28-30].

A remarkable example of these conflicts is the classification of *X. fuscans aurantifolii *[26,27], also known as *X. axonopodis *pv. "*aurantifolii*" [2,6,18,31]. This taxon was originally identified as part of the DNA hybridization homology group "*X. axonopodis*" [6], but after its differentiation from other xanthomonads by DNA sequence-based molecular techniques, production of water-soluble brown pigment and host range, it was designated as *X. fuscans *[26]. However, when these traits/methods were examined, none of them could individually differentiate *X. fuscans *from other pathovars within *X. axonopodis *[18,31]. DNA-DNA reassociation assays, in turn, have differentiated *X. fuscans *from *X. axonopodis, X. campestris *and *X. citri *[2,26,27]. Additional host-range evidence has also been used to support the designation *X. fuscans*, separated from *X. axonopodis *and *X. citri. Phaseolus vulgaris *and *Citrus *spp. are infected by *X. fuscans *pvs. *fuscans *and *aurantifolii*, respectively, but are not infected by either *X. axonopodis *or *X. campestris. Citrus *spp., on the other hand, is also infected by *X. citri *[1]. However, host range is usually a criterion to separate pathovars and not species. This example underscores the importance of a solid taxonomic classification with a phylogenetic basis.

Molecular phylogenetics has played an important role in the classification of the genus. Single locus analyses, including the use of 16S-23S rDNA spacers, the 16S rRNA gene and the DNA gyrase *gyrB *[32-35], generally agree with standing nomenclature but with low resolution below the species level. MLSA including sequences of protein-coding genes *dnaK, fyuA *and *rpoD *[31], has significantly extended previous results. In general, MLSA results suggest that *X. citri *and *X. fuscans *are closely related species and should be considered as a single species based on their 98.34% similarity in the proteins encoded by *dnaK, fyuA, gyrB *and *rpoD *[31]. Recently, a phylogenomic approach was applied to resolve the phylogenetic relationships within the genus [11], although this work did not explore the phylogenetic distances between strains, and did not include sequences from *X. axonopodis *species. The general structure of the genus agreed with the standing nomenclature.

The use of genomic sequences as the basis for species delimitation has been explored as a new standard in bacteria in replacement of DNA-DNA hybridization [36,37], particularly based on metrics such as the ANI (Average Nucleotide Identity) [38]. The correspondence between DNA-DNA hybridization and sequence similarity has been exploited in *Xanthomonas *for the establishment of clades and species [31], but full genomic sequences have not been used so far for the resolution of the "*X. axonopodis*" clade (this is, including close relatives such as *X. fuscans *and *X. euvesicatoria*). Phylogenomic methods extend the analysis of primary sequence data from one or few loci (usually no more than twenty) to hundreds or thousands of loci at the same time, alleviating the problem of incongruence between characters [39,40]. Here, we present a phylogeny of the genus based on seventeen complete and draft genomes, including five genomes from the "*X. axonopodis*" clade. We identified the orthologous genes and performed the phylogenetic inferences using a new library called Unus, which is briefly described here.

## Results

### The automated selection of orthologous genes is consistent with manual selection

In order to compare a typical literature-based selection of genes for phylogenetic reconstruction in bacteria with the Unus automated method, using 989 genes in the genomes listed in Table 1, we evaluated the presence of the housekeeping genes used by AMPHORA [41]. We found that several of these genes were absent in the draft genomes Xfa1, Xfa0 and Xvm0. In addition, in-paralogs (*i.e*., duplicated genes) were detected in the genome of XooK for several ribosomal proteins (large subunit; *rplA, rplC, rplD, rplE, rplF, rplN*) and were therefore discarded. This is possibly due to errors in the genome sequence, given that these genes are usually present as a single copy. Importantly, the absence of *rpl *genes in the XooK genome suggests that ribosomal proteins (from both the small and the large subunits) were located at mis-assembled regions of the genome sequence. Genes employed in the genus-wide analysis and used by AMPHORA include *dnaG, nusA, pgk, pyrG, rplM, rplP, rplS, rplT, rpmA, rpoB, rpsB, rpsC, rpsE, rpsI, rpsK, rpsM *and *rpsS*. Also, five out of the seven genes used by Pieretti *et al. *[42] (*gyrB, recA, dnaK, atpD *and *glnA*) were found in the constructed Orthology Groups (OG), while other two (*groEL *and *efp*) seemed to be absent in the draft genome of Xfa1. This underscores the importance of a flexible selection criterion of orthologous genes in a determined group of taxa, especially with unfinished genomes. A previous MLSA conducted by Young and collaborators [31] employed four protein-coding genes included in the previous lists plus the *tonB*-dependent receptor *fyuA*, also present in our selection. Another MLSA recently performed by Bui Thi Ngoc *et al. *[21] used the genes *atpD, dnaK, efP *and *gyrB*, all of which were present in our dataset. These data suggest that the automated selection using Bit Score Ratio (BSR) is in agreement with the classical selection of genes for phylogenetic studies. Therefore, some of the genes selected in this study can be used for future phylogenetic reconstructions.

**Table 1:** Genomes used in this study

(Sub)species	Pathovar	Strain	**Abbr**.	Caused disease	Database entry	Reference
*X. campestris *(Pammel 1895) Dowson 1939 emend. Vauterin *et al *1995	campestris	BCCM/LMG 8004 *^(1)^	Xcc8	Crucifer black rot	NCBI GI:66766352	[43]

*X. campestris *(Pammel 1895) Dowson 1939 emend. Vauterin *et al *1995	campestris	ATCC 33913^T ^*^(2)^	XccA	Cabbage black rot	NCBI GI:21166373	[44]

*X. campestris *(Pammel 1895) Dowson 1939 emend. Vauterin *et al *1995	campestris	B100 *^(3)^	XccB	*Brassica *black rot	NCBI GI:188989396	[45]

*X. campestris *(Pammel 1895) Dowson 1939 emend. Vauterin *et al *1995	armoraciae	756 C *^(4)^	Xca7	*Brassica *leaf spot	JCVI CMR org:Xca	Unpublished

*X. citri *subsp. *citri *(*ex *Hasse 1915) Gabriel *et al *1989	N/A	306	Xci3	Citrus canker A	NCBI GI:21240774	[44]

*X. fuscans *subsp. *aurantifolii *Schaad *et al *2007 *^(5)^	N/A	ICPB 11122	Xfa1	Citrus canker B	NCBI GI:292601741	[11]

*X. fuscans *subsp. *aurantifolii *Schaad *et al *2007 *^(5)^	N/A	ICPB10535 *^(6)^	Xfa0	Citrus canker C	NCBI GI:292606407	[11]

*X. euvesicatoria *Jones *et al *2006	N/A	85-10	Xeu8	Pepper and tomato bacterial spot	NCBI GI:78045556	[46]

*X. axonopodis *Starr and Garces 1950 emend. Vauterin et al 1995	manihotis	CIO 151 *^(7)^	XamC	Cassava Bacterial Blight	Not in public databases	Unpublished

*X. vasicola *Vauterin *et al *1995	vasculorum	NCPPB 702 *^(8)^	XvvN	Sugarcane gumming disease	NCBI GI:257136567	[47]

*X. vasicola Vauterin et al *1995	musacearum *^(9)^	NCPPB 4381 *^(10)^	XvmN	Banana bacterial wilt	NCBI GI:257136682	[47]

*X. vasicola Vauterin et al 1995*	musacearum *^(9)^	unknown	Xvm0	Banana bacterial wilt	JCVI CMR org: ntxv01	Unpublished

*X. oryzae *(*ex *Ishiyama 1922) Swings *et al *1990 emend. van der Mooter and Swings 1990	oryzae	KACC 10331*^(11)^	XooK	Rice bacterial blight	NCBI GI:58579623	[48]

*X. oryzae (ex Ishiyama 1922) Swings et al 1990 emend. van der Mooter and Swings 1990*	oryzae	MAFF 311018 *^(12)^	XooM	Rice bacterial blight	NCBI GI:84621657	[49]

*X. oryzae (ex Ishiyama 1922) Swings et al 1990 emend. van der Mooter and Swings 1990*	Oryzae	PXO99^A *(13)^	XooP	Rice bacterial blight	NCBI GI:188574270	[50]

*X. oryzae (ex Ishiyama 1922) Swings et al 1990 emend. van der Mooter and Swings 1990*	oryzicola	BLS 256	XocB	Rice bacterial streak	NCBI GI:94721236	Unpublished

*X. albilineans *(Ashby 1929) Dowson 1943 emend. van der Mooter and Swings 1990	N/A	GPE PC73 *^(14)^	XalG	Sugarcane leaf scald	NCBI GI:283472039	[42]

The (Sub)species column contains the accepted name of the bacterium. Alternative names may exist. The listed diseases may be known with different names or in additional hosts. The diseases names and hosts stand as designated in the publication of the genome (rightmost column) or in [8] where unpublished. *(1) Spontaneous rifampicilin-resistant strain derived from NCPPB 1145 (StrainInfo 23435). *(2) Type strain of the species, StrainInfo 23352. *(3) Sm^r ^derivative of the wild-type strain DSM 1526 [51], StrainInfo 157307. *(4) Wild-type isolate by Anne Alvarez [52]. *(5) In this study we show that this name should be considered a later heterotypic synonym of *X. citri *as previously suggested [18,31]. *(6) IBSF 338, StrainInfo 545646. *(7) CIO, CIAT-ORSTROM (now IRD) *Xanthomonas *collection, Biotechnology Research Unit, Cali, Colombia [53]. *(8) CFBP 7169 or LMG 8710, StrainInfo 26110. *(10) Isolated from banana by Valentine Aritua, not registered in StrainInfo. *(11) CFBP 7088, StrainInfo 559506. *(12) StrainInfo 373786. *(13) 5-azacytidine-resistant derivative of PXO99, collected by Mew and collaborators [54]. *(14) CFBP 7063, StrainInfo 843129.

**Figure 1:**
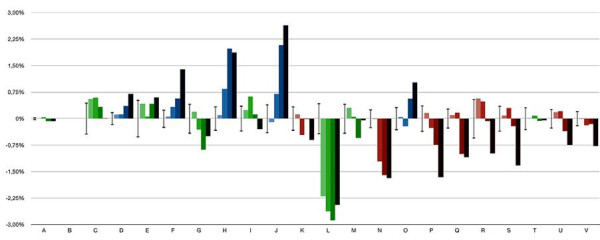
**Enrichment of COG categories in several OG sets**. The ordinates axis shows the COG categories. The subordinate axis accounts for the difference between the representation of the category in the OG set and the representation of the category in the reference genome Xeu8. Each bar represents a category in a given OG set. Sets from lighter to darker are: **Xeu8 **genes discarding in-paralogs; ***X. axonopodis *clade**, including Xeu8, XamC, Xci3, Xfa0 and Xfa1; **No-XalG**, including all the genomes in the study but XalG; ***Xanthomonas***, including all the genomes in the genus *Xanthomonas*. Error bars indicate one positive and one negative standard deviation calculated as described in the methods. Categories increasing in representation at wider taxonomical ranges are hued **blue**. Categories decreasing in representation at wider taxonomical ranges are hued **red**. Other categories are hued **green**.

The COG classification for the employed genes (Additional file 1) was compared among sets of genes obtained from automated selections at different taxonomical levels within the genus (Figure 1). COG categories related to central metabolism and ribosomal proteins presented a tendency to increase in representation (relative to other COG categories), as genomes from a wider taxonomical range were included (blue bars in Figure 1). Together, these categories covered 27% of the COG-classified genes and included genes that are frequently used for phylogenetic reconstruction. On the other hand, a reduction in the relative representation when including a wider taxonomical range of genomes was observed for categories related to peripheral metabolism and poorly characterized proteins (red bars in Figure 1). These categories covered 36.9% of the COG-classified genes and included clade-specific genes (without detectable orthologs in distant relatives) as well as genes absent in *X. albilineans*, which presents a notable genome size reduction [42]. Pieretti and collaborators identified 131 ancestral genes potentially lost by pseudogenization or short deletions in *X. albilineans *and 480 potentially lost by both *X. albilineans *and *Xylella fastidiosa *[42]. Most of the COG-classified genes putatively lost in *X. albilineans *or both *X. albilineans *and *Xylella fastidiosa *(56.2% and 56%, respectively) can be classified within these COG categories. The same tendency to increase in relative representation when increasing the number of taxa was displayed by genes without an assigned COG category (data not shown). The only category significantly impacted by discarding the in-paralogs was category L (replication, recombination and repair). This category covers 8.2% of the COG-classified genes, and 83.2% of those discarded by paralogy, suggesting frequent duplications of genes implicated in these processes. Putative transposases and inactive derivatives represent 76% of the discarded genes.

### Phylogeny of the genus *Xanthomonas*

Our phylogenetic analysis was based on 989 OG (1,084,777 bp, Additional file 2), which included all markers used in previous *Xanthomonas *phylogenetic analyses. Both, the Maximum Likelihood tree and the Bayesian consensus tree reconstructed the same well-supported topology, with bootstrap supports of 100% for all the nodes (out of 1,001 replicates). The same relationships were also obtained with Maximum Parsimony (bootstrap support of 100% with 1,000 replicates).

**Figure 2:**
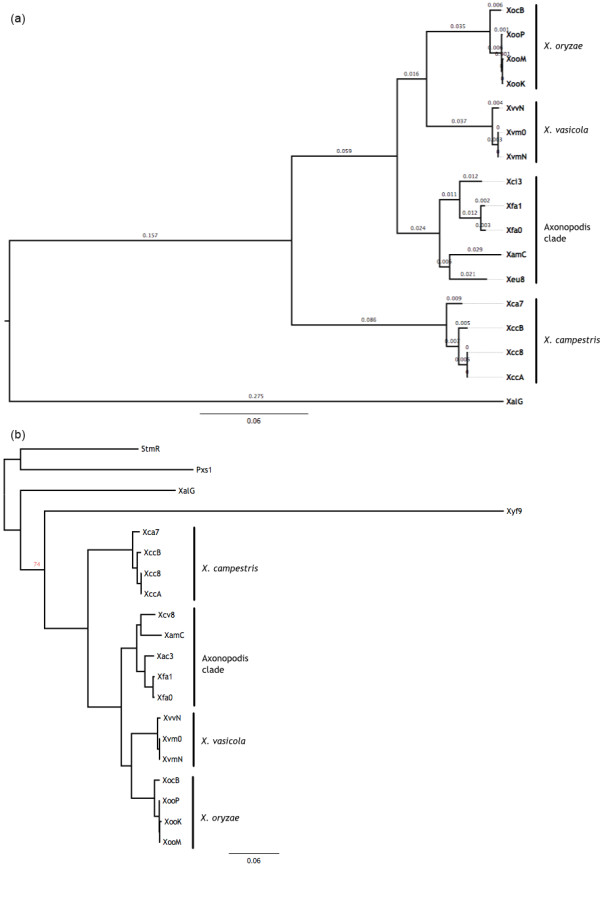
**Genome-based phylogeny of *Xanthomonas***. Consensus phylogenetic tree of strains of **(a) ***Xanthomonas *based on the 989 OGs, with *X. albilineans *as an outgroup and **(b) ***Xanthomonas *and some genomes from the close relatives *Pseudoxanthomonas, Xylella *and *Stenotrophomonas *based on 228 identified using the BSR automated method. Branch lengths are according to the ML-based inference. All nodes were inferred to have a bootstrap value of 100% in 100 samplings. All nodes were inferred to have posterior probability of 1.0 based on 1,001 trees sampled from the posterior distribution in the Bayesian inference, with identical topology. Numbers above each branch indicate the branch length estimated as the proportion of expected changes per site.

A total of four clades were obtained in the phylogenomic reconstruction. The first clade includes *X. oryzae*, the second comprises *X. vasicola*, the third one groups together *X. fuscans, X. euvesicatoria *and *X. axonopodis*, and the fourth clade contains *X. campestris *(Figure 2a). These results agree with previous phylogenies of the genus [11,17,35,42]. In order to further advance on the knowledge of the ancestral relationships of the genus *Xanthomonas*, and in particular the species *Xylella fastidiosa*, we performed a new analysis including three additional genomes in the *Xanthomonadaceae *family: *Xylella fastidiosa *str. 9a5c (GenBank entry AE003849.1), also a plant pathogen, but strictly transmitted by insect vectors; *Pseudoxanthomonas suwonensis *str. 11-1 (GenBank entry CP002446.1), a bacterium isolated from environmental samples but more commonly found in contaminated ones; and *Stenotrophomonas maltophilia *str. R551-3 (GenBank entry NC_011071.1), a common soil colonizer which has also been reported as a human opportunistic pathogen. These species are hereafter termed Xyf9, Pxs1 and StmR, respectively. This new analysis was based on a collection of 228 genes automatically compiled by the Unus library using Bit Score Ration (BSR). The resulting phylogeny revealed that the genus *Xanthomonas *is not monophyletic, with *Xylella fastidiosa *as its sister clade. *X. albilineans *should be placed in an independent genus in order for the taxonomy to match the phylogeny of the group (Figure 2b), as previously noted [42]. This result differs from that presented by Pieretti and collaborators, based on seven housekeeping genes [42], where *X. albilineans *and *X. fastidiosa *form a single clade ancestral to all other *Xanthomonas*.

### Genome evolution: gains and losses

**Figure 3:**
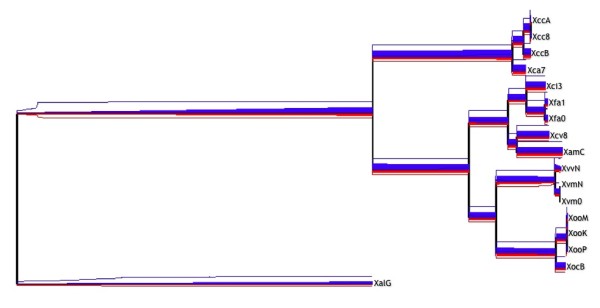
**Genomic gains and losses in the genus *Xanthomonas***. Gains (**red**) and losses (**blue**) predicted in genomic regions along branches of the phylogenetic tree of *Xanthomonas*. The width of red and blue lines are proportional to the average detected genomic gains and losses, respectively, and a 95% confidence interval is presented as red and blue lines above and below solid regions, respectively.

The high number of pseudogenes and lost regions in *X. albilineans *suggests a reductive genome evolution in this species [42]. This information, together with the position of the taxon in previous phylogenies [11,42] and the reduced size of the close relative *Xylella fastidiosa *[55], could indicate either a reduced genome as the ancestral condition in the *Xanthomonas *genus or independent genome reductions in *Xylella fastidiosa *and *X. albilineans*. Pieretti and collaborators provide strong evidence supporting the latter hypothesis [42]. However, the enrichment of phage-related regions in the *Xylella *genomes, as well as the presence of multiple Insertion Sequences (IS) in *Xanthomonas *reveal very active mobile elements in the *Xanthomonadales *order [56]. To determine whether this reductive tendency extends to other genomes of the genus, we employed GenoPlast [57] for the detection of ancestral genomic gains and losses. The results (Figure 3 and Additional file 3) revealed that all the tip nodes in the *X. oryzae *species present net genomic losses compensated by genomic gains in ancestors of the species (*i.e*., internal nodes 20 and 24, as labeled in Additional file 3). Interestingly, the three genomes of the species *X. vasicola *presented large genomic gains (between 12.78% and 15.19% of the regions) after genomic losses exhibited by the most recent ancestral node of the species (11.47% of the regions). This level of genomic losses is almost twice as large as that exhibited by *X. albilineans *(5.92%), suggesting that the *X. vasicola *genomes are very dynamic, while maintaining a genome size comparable to other species in the genus.

### Gene clusters and detection of putative gene transfer by orthology groups

**Figure 4:**
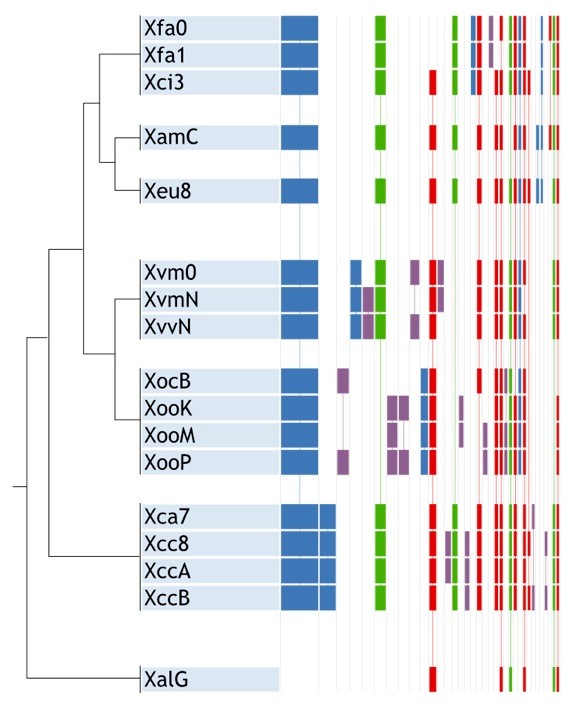
**Groups of orthology among seventeen *Xanthomonas *genomes**. A cladogram of phylogenetic relationships inferred here is shown on the left. Coloured boxes represent groups of orthologs as detected by OrthoMCL. Each column represents a pattern of presence/absence, and the width of the boxes is proportional to the number of genes showing the given pattern. The colour code is as follows: **blue **for monophyletic patterns involving all the strains on each species (the pattern including all the genomes coloured light blue); **green **for evolutionary changes below the species level; and **red **for patterns involving strains from more than one species and excluding at least one strain of these species. Patterns are ordered by number of genes: columns decrease in number of genes from left to right.

In order to identify the distribution of OGs among taxa within *Xanthomonas*, a second set was constructed using OrthoMCL [58]. Figure 4 depicts the general distribution, clustering by patterns of presence/absence among genomes, regardless of their relatedness. In general, the patterns presented by most of the OGs are monophyletic, as expected (blue columns in Figure 4). However, a few paraphyletic patterns were unexpectedly enriched. Further inspection revealed that most of the OGs in two of the most enriched paraphyletic patterns are clustered in the genomes and preserve synteny. We explored these patterns, and found two clusters of contiguous genes with paraphyletic distributions, suggesting horizontal transference of genetic material.

**Figure 5:**
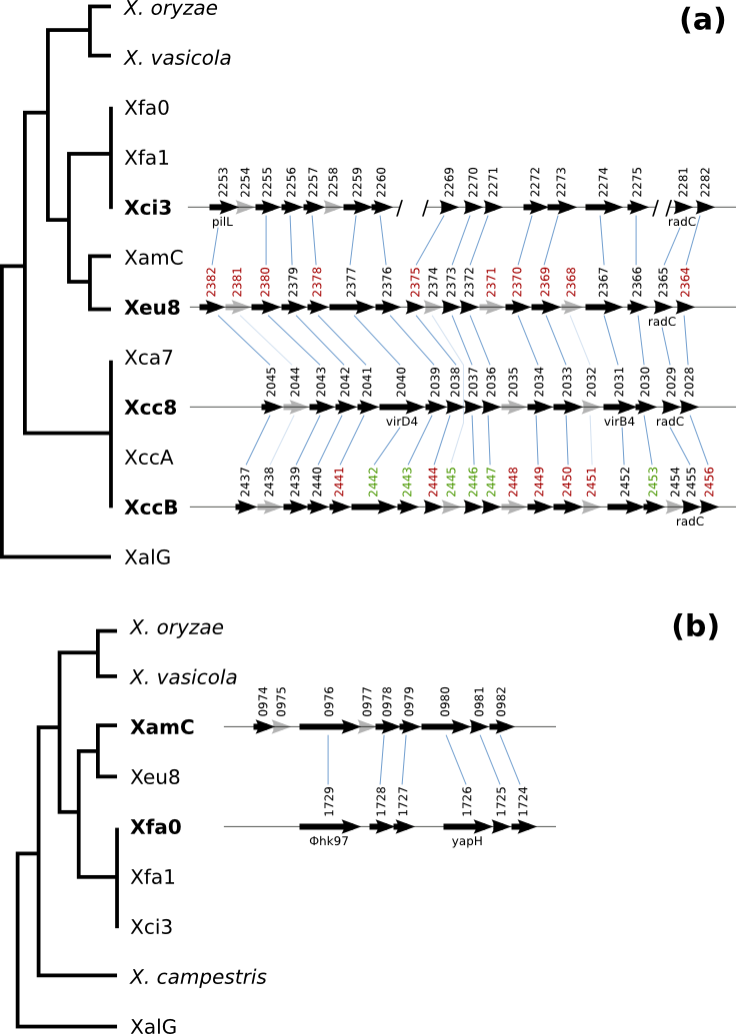
**Clusters of genes identified by patterns of orthology**. The patterns presented in Figure 5 were used for the identification of two clusters of genes potentially displaying cases of LGT. Dendrograms on the left are derived from Figure 3a (branch lengths do not represent inferred distances). Detected orthologs are only present in the genomes in bold. **Arrows in black **represent genes in an OG of the highlighted pattern and **grey arrows **represent other genes nearby in the genome. **Blue lines **linking genes indicate inferred orthology. Gene numbers correspond to the last part of the original gene names. Numbers in colours other than black indicate genes with products putatively secreted (**red**) or with transmembrane domains (**green**). The clusters are **(a) **one including a wrongly annotated pathogenicity-related gene (*yapH*) and a phage gene (Φ-hk97); and **(b) **one possibly related to the type IV secretion system.

The first cluster (Figure 5a) is present in Xci3, Xeu8, Xcc8 and XccB, but absent in other genomes of *X. campestris*, in *X. axonopodis *and in *X. fuscans*. Similar genes were also found in *Pseudomonas aeruginosa, Salmonella enterica *and other species of the genera *Pseudomonas, Salmonella *and *Acidovorax *(Additional file 4). This cluster is mainly composed of putative secreted and membrane proteins, with few characterized orthologs. In *Xanthomonas*, only three of those genes have been characterized. The first two code for VirD4 and VirB4, which are proteins implicated in protein secretion by the Type IV secretion system in several bacteria, including *Helicobacter, Agrobacterium *and *Bartonella *[59,60]. The third codes for RadC, a protein involved in DNA repair. The gene at the locus XCV2366_1 from Xeu8 presents homology with the oxidoreductase DbsA, an important protein for oxidative folding of disulphide-bonded proteins in Gram-negative bacteria [61]. Only nine out of the nineteen genes in this cluster present a G+C content at least one standard deviation distant from the average for the coding regions within the Xeu8 genome (64.66 ± 3.91%). The values of Codon Adaptation Index (CAI) for the seventeen genes in the cluster were similar to the values obtained for other regions of the genome. The distribution of this cluster along the genus suggests flow of genetic material between different pathovars of *Xanthomonas*. However, G+C content and CAI analyses failed to relate this cluster to LGT. Furthermore, LGT regions predicted by AlienHunter [62] do not cover more than one gene in this region in any of the analysed genomes (data not shown). Interestingly, in all the genomes, predicted LGT regions surround the cluster at distances from one to eight Kbp.

The second cluster (Figure 5b) is present in XamC and Xfa0 but not in Xfa1, despite the high genome-wide similarity presented between Xfa1 and Xfa0 (Figure 2a). The classification of putative homologs of the genes in this cluster (see methods) revealed that it is mainly composed of sequences similar to proteins in *Escherichia coli, Siphoviridae, Stenotrophomonas *sp. SKA14, *Salmonella enterica *and *Pseudomonas aeruginosa *(Additional file 5). Moreover, members of the *Siphoviridae *viral family are known to be *Pseudomonas *and *Xanthomonas *phages, suggesting the presence of virus-mediated LGT. We cannot attribute the pattern to the mixture of chromosomal and plasmidic DNA in draft genomes (XamC and Xfa0), because none of the sequences presented similarity with genes in *Xanthomonas *plasmids. Note that the gene at the locus XAUC_17260_1 (Xfa0:1726 in Figure 5b) was originally annotated as *yapH*, but its product is a large protein of 1231 aa in Xfa0 and 1482 aa in XamC, putatively xenologous with a component of a phage tail (group COG4733 in the COG database). Two genes in the cluster (XamCg00977 and XamCg00978) presented a G+C content more than one standard deviation below the mean of the coding sequences in the XamC genome (*i.e*., 64.82 ± 3.31%), and a low CAI with respect to the whole predicted coding sequences (0.516 and 0.486, respectively). The other seven genes in the cluster presented average features, which would have precluded their identification as units potentially under LGT.

## Discussion

The results of the genome-based phylogenetic reconstruction suggest that certain changes should be considered in the nomenclature of the *Xanthomonas *genus. For instance, *X. fuscans *was recently proposed as a new species [27], but here we show that it should be considered as a later heterotypic synonym of *X. citri*, as previously suggested [18,31]. Other clades in the standing bacterial nomenclature [63] within the *Xanthonomonas *genus were consistent with the phylogenetic reconstruction. Nevertheless, we observed a paralogy in the genus *Xanthomonas *when *Xylella fastidiosa *was included with *X. albilineans *outside the *Xanthomonas *group. Our results suggest that *X. albilineans*, probably along with other early-branching *Xanthomonas*, should be considered for a new genus designation. However, the relationships between *X. albilineans, Xylella *and the other *Xanthomonas *remain unclear. Another shared feature between *Xylella fastidiosa *and *X. albilineans *is the reduced genome. The reductions in these genomes were previously shown to be due to independent events [42]. Here we show evidence suggesting that reductive genome evolution could also affect other clades in the genus such as *X. vasicola*.

The phylogenetic relationship between *X. albilineans, Xylella fastidiosa *and the rest of the taxa in the genus *Xanthomonas *is not clear. The genome of *X. albilineans *is part of the "early-branching species" [7], a group of species including *X. albilineans *and *X. sacchari *previously found to be basal in the phylogeny of the genus [7,35]. The species is also a member of the "*hyacinthii*" group, a group of species with major differences in the 16S-23S rDNA Intergenic Spacer (ITS) with respect to the other members of the genus [32]. Pieretti and collaborators [42] suggested that *Xylella *and *X. albilineans *form a monophyletic clade, which is basal to the rest of *Xanthomonas*. This is based on a Maximum Likelihood analysis with seven housekeeping genes. Our analyses with over two hundred genes suggest that *X. albilineans *is basal to *Xylella *and the rest of taxa in the genus *Xanthomonas*. Neither of the analyses obtains a good support value for these nodes. The most straightforward explanation for this is that certain regions of the genome support one topology and certain others support the second one. This could be due to a considerable number of LGT in these genomes. Alternatively, it could be due to the large amount of changes accumulated in *Xylella fastidiosa*, as revealed by the length of the corresponding branch (Figure 2b).

The phylogenetic tree presented in Figure 2a displays identical topology and similar relative branch lengths as inferred by different optimality criteria (Maximum Likelihood, Bayesian Inference, Maximum Parsimony). The tree supports monophyly in the species *X. campestris, X. oryzae *and *X. vasicola*. The clade "*X. axonopodis*" contains the species *X. fuscans, X. citri, X. axonopodis *and *X. euvesicatoria*. However, the lower coverage in terms of sequenced genomes of these species makes it difficult to support any further observation beyond the close relatedness within the clade with respect to other species.

Interestingly, the phylogeny displays a close relationship between the species *X. fuscans *and *X. citri*. In order to compare their similarity in the same framework of MLSA performed for other species of *Xanthomonas *(*e.g*., [31]), we constructed a matrix containing 989 loci employed for the phylogenetic inference (Table 2). According to the resulting matrix, a similarity threshold of 99% can differentiate bacteria recognized as belonging to the different pathovars (except in *X. vasicola*, for which pathovars *vasculorum *and *musacearum *display a similarity above 99%, possibly due to non-chromosomal sequences). All the species with currently accepted names [63] have similarities above 97%. This value (in accordance with previous MLSA calibrations [31]) also differentiate species outside the *X. axonopodis *clade, but fails to differentiate *X. fuscans *and *X. citri*, suggesting that the two pathovars conform a single species as previously suggested [18,31]. This is also supported by the likelihood distances between these two taxa (Figure 2a, Table 2). Accordingly, we recommended that the species *X. fuscans *be regarded as a heterotypic synonym of *X. citri*.

**Table 2:** Similarity matrix between genomes

Genome	XccA	XccB	Xca7	Xci3	Xfa1	Xfa0	Xeu8	XamC	XvvN	XvmN	Xvm0	XooK	XooM	XooP	XocB	XalG
**XccA**	**100.00%**															

**XccB**	**99.08%**	**100.00%**														

**Xca7**	98.17%	98.15%	**100.00%**													

**Xci3**	87.81%	87.80%	87.88%	**100.00%**												

**Xfa1**	87.85%	87.77%	87.84%	97.63%	**100.00%**											

**Xfa0**	87.81%	87.73%	87.79%	97.59%	**99.51%**	**100.00%**										

**Xeu8**	87.93%	87.85%	87.92%	95.97%	95.82%	95.77%	**100.00%**									

**XamC**	87.97%	87.89%	87.96%	95.38%	95.25%	95.22%	95.80%	**100.00%**								

**XvvN**	87.54%	87.47%	87.52%	92.48%	92.44%	92.39%	92.40%	92.11%	**100.00%**							

**XvmN**	97.60%	87.54%	87.59%	92.52%	92.47%	92.43%	92.48%	92.14%	**99.36%**	**100.00%**						

**Xvm0**	87.51%	87.42%	87.47%	92.44%	92.44%	92.37%	92.39%	92.12%	**99.34%**	**99.97%**	**100.00%**					

**XooK**	87.32%	87.17%	87.31%	92.29%	92.24%	92.21%	92.26%	91.94%	93.51%	93.58%	93.48%	**100.00%**				

**XooM**	87.36%	87.34%	87.41%	92.31%	92.27%	92.24%	92.30%	91.99%	93.53%	93.59%	93.51%	**99.91%**	**100.00%**			

**XooP**	87.43%	87.35%	87.40%	92.32%	92.26%	92.23%	92.29%	91.99%	93.53%	93.58%	93.50%	**99.88%**	**99.85%**	**100.00%**		

**XocB**	87.41%	87.32%	87.39%	92.37%	92.31%	92.27%	92.34%	92.03%	93.57%	93.62%	93.54%	98.78%	98.78%	98.80%	**100.00%**	

**XalG**	78.52%	78.43%	78.54%	78.47%	78.41%	78.38%	78.44%	78.62%	77.96%	78.04%	77.95%	77.94%	78.02%	78.06%	78.02%	**100.00%**

The 989 loci employed for phylogenetic inference were used to generate a similarity matrix between genomes. Values between 96-99% of similarity are highlighted in light **grey**. Values above 99% similarity are in bold.

Several robust methods for the identification of orthology, multiple sequence alignments and phylogenetic inferences have recently been developed (reviewed in [64]). However, a common flexible framework for their joint application in specialized phylogenetic studies and MLSA in general is still required. The BioPerl libraries, including the Bio::Phylo package [65,66], provide valuable tools for the automation of analyses, but the connections between different steps are often not automated, making them time-consuming. Unus allows the execution of complete workflows in phylogenomics within a single interface, and its current functionalities and limitations underscore the need for a fully structured platform in the field, such as those available for other branches of genomics.

We compared the automatically selected OGs for the phylogenetic assessment with several lists of genes manually compiled. These comparisons indicated that, depending on the genome coverage and annotation of the drafts employed, our analyses broadly agree in the selection of OGs with those utilized previously for phylogenetic inference. Furthermore, the functional distribution of the automatically selected genes exhibits the expected behaviour at different taxonomical levels. Selections on broader taxonomical levels exhibit a larger representation of genes implicated in central-metabolism, while the proportion of clade-specific genes augments in narrower taxonomical levels.

The analysis of the distribution of COG categories shows that central metabolism and ribosomal proteins are favoured when comparing distant genomes, as they are in phylogenetic studies based on one or few loci. Genes in these categories are better suited than genes in other COG categories or unclassified genes because of two characteristics that are important for phylogenetic assessment. Firstly, genes implicated in central-metabolism and ribosomal genes are usually of single-copy. Genes with in-paralogs are normally avoided in phylogenetic inferences given the difficulty in identifying corresponding genes in sets of paralogy [67], despite some efforts to include them in phylogenetic analyses (*e.g*., [68]). Secondly, these genes are often present even in genomes from loosely related organisms. Although phylogenetic reconstructions based on gene content have proven successful (*e.g*., [69]), it is hard to achieve high resolution below species and it is not possible with incomplete draft genomes.

Additional genes suitable for phylogenetic analyses were detected through automated identification of orthologs, allowing a higher resolution among closely related taxa. These genes are usually not included in MLSA, although they can add important information about relationships within the group. For closely related bacteria (such as the *X. oryzae *pv. *oryzae *strains), the importance of such additional information resides on the low variability among genomes. Therefore, the option to select orthologs without *a priori *knowledge of the genes that will be included, allows for flexibility in terms of data availability, as well as the obtention of optimized phylogenetic resolution at any taxonomic level under study.

A previous study [42] suggested a reductive evolution in the genome of *X. albilineans*, revealed by the small genome (3.77 Mbp) and the high putative pseudogenization. We present evidence supporting the hypothesis that the reductive genome evolution occurs along the genus, and is not restricted to the species *X. albilineans*. In our analyses, the species *X. albilineans *effectively revealed large genomic reductions, but even larger reductions were presented by the species *X. vasicola*, with recent genomic gains only detected on tip nodes, suggesting a reductive evolution tendency followed by the acquisition of genomic regions. The genomic gains on tip nodes can be partly explained by the inclusion of non-chromosomal material in the draft genomes of *X. vasicola*, although this result was not found in other draft genomes in the study that have non-chromosomal material, such as XamC. An alternative explanation is that genomic gains have arisen by recent genetic exchange with other bacteria, as previously suggested for *X. vasicola *[47]. However, the large ancestral losses cannot be explained by means of the incompleteness of the genomes, and may reflect an ancestral genomic reduction in the species. The size of the regions involved in such events, and whether they affect restricted functional categories of genes or random regions, is still to be determined.

We identified two clusters of genes with paraphyletic distribution, suggesting lateral gene transfer. One of the clusters, present in *X. campestris *and the "*X. axonopodis*" clade, exhibits interesting functional relationships with the Type IV Secretion System (T4SS), while most of the genes are annotated as coding for either putative secreted or membrane proteins. Identification of LGT events based only on intrinsic features such as the G+C content and the CAI would fail to identify both clusters, showcasing the usefulness the phylogenetic distribution of orthologs as a complement for the prediction of putative LGT events.

## Conclusions

Currently, phylogenomic methods are finding a privileged place in phylogenetic inference and evolutionary studies, yet common frameworks for the flexible automation of workflows are not widely available. Here we used Unus, a package developed to facilitate the execution of phylogenetic workflows, to explore the phylogenetic structure of the genus *Xanthomonas*. We recovered a strongly supported phylogeny in accordance with previous results and high resolution in the closely related genomes of *X. oryzae*. The results also provide evidence for the reconsideration of the *X. fuscans *species, clarify relationships between *X. citri, X. axonopodis *and *X. euvesicatoria*, and show that the genus *Xanthomonas *is not a monophyletic clade. Our results allowed us to identify several interesting features in the evolution of *Xanthomonas*, including two large putative lateral gene transfer events, which would have been hard to detect by means of G+C content deviation or Codon Adaptation Index. We also detected evidence of an evolutionary tendency towards a reduction in genome size in at least two clades of the genus.

## Methods

### *Xanthomonas *genomes

Seventeen *Xanthomonas *genomes were used in this study (Table 1). The names employed follow the list of prokaryotic names with standing nomenclature (LPSN) [63], although several additional names may exist in the scientific literature. Whenever possible, the strains have been tracked to the corresponding StrainInfo entry [70], in order to ease the resolution of strains deposited in different collections. Gene and gene product predictions were downloaded together with the genomes from NCBI (when available) and JCVI websites, except for the genome of *X. axonopodis *pv. *manihotis *str. CIO151 (unpublished), for which coding sequences (CDS) were predicted using Glimmer 3 [71] trained with the *X. euvesicatoria *str. 85-10 CDS [46]. All the genomes are referred to as stated in the abbreviation column in Table 1.

### Generation of Unus, a new library for the execution of phylogenomic workflows

**Figure 6:**
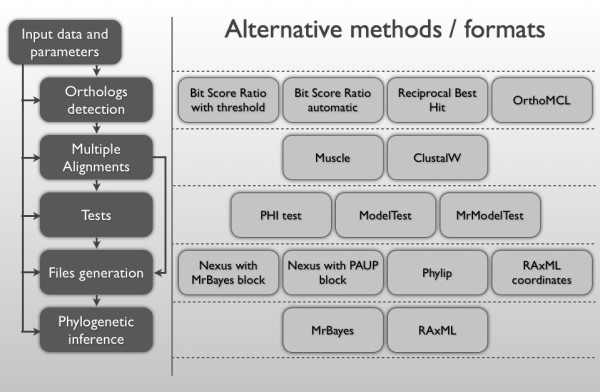
**Workflows executable with the Unus libraries**. The workflow on the left depicts the multiple steps allowed by the Unus library. Each step has multiple alternative methods or formats listed on the right side of the diagram.

Unus is a Perl library that enables the easy execution of phylogenomic workflows including the detection of groups of orthologous genes, batch alignment of sequences, generation of files in a variety of formats and integration of accessory tests for recombination and models of evolution. The various possible workflows the user can go though in order to obtain a phylogenomic inference of the group of bacteria of interest are depicted in Figure 6. Fourteen Perl modules integrating the Unus package are available for download and code browsing at http://github.com/lmrodriguezr/Unus/. Figure 6 summarizes the different pipelines implemented with Unus and alternative programs that can be used.

### Detection of orthologous groups

For the detection of Orthologous Groups (OG), we used the distribution of the Bits Score Ratio (BSR), a BLAST-based metric [72] essentially as previously described [10]. Briefly, the BSR is defined as the proportion of the Bit Score of the alignment of the query sequence and the subject sequence, and the Bit Score of the alignment of the query sequence with itself (*i.e*., the maximum Bit Score for a given query). The histogram is usually bimodal (Additional file 6), and Unus detects the valley of the distribution as the threshold to accept a hit for each paired comparison. To avoid spurious results in distributions with shallow valleys or with no evident valley, the threshold for three distributions was set as the average threshold (as calculated for the other paired comparisons). This method accounts for the problems previously observed when considering the best hit only [73,74], as in widely used methods such as the BLAST Reciprocal Best Match (RBM), also implemented for comparison (see Additional file 7 for the annotated pseudo-code).

### Phylogenetic inference

Multiple sequence alignments were performed using MUSCLE [75] on each detected OG. Alignments were discarded when a strong signal of recombination was detected in the Phi test [76], *i.e*., *p*-value ≤ 0.01 under the null model of no recombination. Phylogenetic inference based on whole genomes used Maximum Likelihood (ML) optimality criterion, as implemented in RAxML v7.2.6 [77,78] with the GTRCAT option, which takes the GTR model of nucleotide substitution, plus an approximation of the Gamma model of rate heterogeneity into account. Branch support was assessed using bootstrap sampling as previously reported [11]. Analyses were performed with each gene in a separate partition to which an independent model of evolution was applied. The resulting ML phylogeny was compared with the consensus topology obtained from Bayesian Inference (BI) [79,80], with exploration of parameters using the Metropolis-Coupled Monte Carlo Markov Chain (MC3) algorithm with one million generations, as implemented in MrBayes v3.1.2, sampling a tree every 1,000 generations. The log-likelihood scores of sampled points were plotted against generation time to determine when the chain became stationary. All sample points prior to this (300,000 trees) were discarded as burn-in samples. Data remaining after discarding burn-in samples were used to generate a majority rule consensus tree, where percentage of samples recovering any particular clade represented the posterior probability of that clade. Probabilities ≥ 95% were considered indicative of significant support. Branch lengths of the consensus tree were estimated by maximum likelihood [81]. We performed additional phylogenetic reconstructions using Maximum Parsimony (MP) using the PAUP* package v4.0b10 [82]. MP trees were obtained in an equal weighted heuristic search with tree-bisection-reconnection (TBR) branch swapping. The consensus tree was calculated using majority rule. Bootstrap (1,000 replicates, heuristic search TBR branch swapping) was used to assess support for each node. A similarity matrix of all the concatenated sequences was prepared using the DNADIST program of the PHYLIP package [77] using Kimura distance [83], in order to compare the distances within the "*X. axonopodis*" clade with previous MLSA.

### Detection of genomic gains and losses

The genomic gains and losses were identified and quantified using GenoPlast [57] with 10,000 burn-in iterations followed by 100,000 additional iterations, 10 iterations between sampling and two independent runs with identical parameters. Analyses were performed assuming a single phylogenetic tree obtained by ML inference. The input multiple alignment was conducted with progressive Mauve [84], and post-processed with the tools for developers of Mauve [85] to first obtain a binary matrix of presence/absence by region, and afterwards a matrix of presence/absence patterns counts. GenoPlast processes this matrix for the calculation of probabilities of ancestral events of genomic gains and losses and implements a model-based method to infer the patterns of genome content evolution by Bayesian inference, assuming a Poisson distribution of genomic gains and losses. The phylogeny inferred here was used as scaffold.

### Assignation of COG functional categories

Homology with entries in the Cluster of Orthologous Groups of proteins (COG) database [86] was determined by BLAST searches [72] against the COG sequences database. The BLAST search was performed using the default filtering algorithm and a minimum quality of alignments defined by a score of at least 250 bits, an identity of 50% of the aligned region or more, and an aligned region comprising 50% of the query gene or more. BLAST results were parsed and filtered using a custom Perl script with the above criteria. The Perl script also mapped the hits to the corresponding COG category, reporting the category or categories for each query sequence. Each set was analysed 1,000 times randomly sampling 75% of the query sequences to calculate the Standard Deviation (SD; Figure 1). For the characterization of OGs, each comprising one gene per genome, only genes present in the genome of *X. euvesicatoria *str. 85-10 were used as representative of the OG.

### Taxonomical distribution of homologous sequences

BLAST searches against the non-redundant protein database of the NCBI (NR) [87] were performed in order to identify the homologs of one or more genes in other organisms, with default parameters and Expect value below 10^-10^. The BLAST result was subsequently parsed with a custom Perl script to extract the organisms, subsequently building a cumulative counts table and mapping these organisms to any fixed taxonomical level using the NCBI's Taxonomy database [87].

## Authors' contributions

LMR participated in the design and coordination of the study, acquired data, carried out the analysis and drafted the manuscript. AG participated in the design and coordination of the study, acquired data and critically revised the manuscript. MLA participated in the design and coordination of the analyses. CS participated in the design and coordination of the study and critically revised the manuscript, while SR participated in the design and coordination and critically revised the manuscript. AB conceived the study, participated in the design and coordination of the study, drafted and critically revised the manuscript. All authors read and approved the final manuscript.

## Supplementary Material

Additional file 1 **COG distribution of different taxonomical ranges**. Raw data graphically presented in Figure 2. Each row corresponds to one COG functional category. Each taxonomical range is represented in two columns, the average and the standard deviation. Click here for file

Additional file 2 **Concatenated sequence alignment and partitions**. ZIP file containing the input alignment in Phylip format (Suppl_file_2.phylip) and the coordinates of the partitions (Suppl_file_2.raxcoords) as employed for the ML phylogenetic analysis in RAxML. Unus automatically generated these files. Click here for file

Additional file 3 **Leaf and ancestral nodes in the GenoPlast events matrix**. Each row corresponds to one node, and each column corresponds to a pattern of regions, as defined by Mauve developers' tools. The first two additional columns contain the node identifier and the node content. Click here for file

Additional file 4 **Species counts in similar sequences of cluster 1**. Species counts within the BLAST hits in NCBI's NR using the genes of Xeu8 in the cluster as query. Click here for file

Additional file 5 **Species counts in similar sequences of cluster 2**. Species counts within the BLAST hits in NCBI's NR using the genes of XamC in the cluster as query. Click here for file

Additional file 6 **Distribution of the BLAST Bit Score (BSR) for several paired comparisons**. The genes of Xeu8 were used as reference to build histograms of BSR values here displayed in logarithmic scale (**blue**). In **purple**, is the distribution by larger windows of values. In **green**, is the automatically selected threshold based on the valley of the distribution. **Discontinuous purple **shows the average threshold, while **grey **indicates four extreme points of the distribution used to evaluate its topology. Click here for file

Additional file 7 **Supplementary methods**. A supplementary text describing methods for the construction of OGs using the Bit Score Ratio with static (BSR-Manual) and dynamic thresholds (BSR-Auto), and the BLAST Reciprocal Best Match (RBM). Click here for file
