# Supplementary material for: Genomes-based phylogeny of the genus Xanthomonas
Source: BMC Microbiol. 2012 Mar 23;12:43. doi: 10.1186/1471-2180-12-43 (PMC3359215; doi:10.1186/1471-2180-12-43)
Supplement: Additional file 1 — COG distribution of different taxonomical ranges. Raw data graphically presented in Figure 2. Each row corresponds to one COG functional category. Each taxonomical range is represented in two columns, the average and the standard deviation. [file 1471-2180-12-43-S1.PDF]

	Xeu8 (ref)	-> SD	Xeu8	-> SD	Xa	-> SD	NoXal	-> SD	Xs	-> SD	I	II	III
A	0,07%	0,04%	0,08%	0,04%	0,11%	0,06%	0,00%	0,00%	0,00%	0,00%	N	Y	N
B	0,00%	0,00%	0,00%	0,00%	0,00%	0,00%	0,00%	0,00%	0,00%	0,00%	N	N	N
C	8,06%	0,45%	8,60%	0,46%	8,64%	0,55%	8,38%	0,66%	8,05%	0,71%	N	N	N
D	1,41%	0,18%	1,52%	0,19%	1,53%	0,23%	1,76%	0,31%	2,11%	0,37%	Y	N	N
E	11,70%	0,53%	12,11%	0,56%	11,76%	0,64%	12,12%	0,81%	12,29%	0,88%	N	N	N
F	2,83%	0,26%	2,89%	0,28%	3,16%	0,33%	3,40%	0,43%	4,22%	0,53%	N	N	N
G	7,80%	0,43%	7,98%	0,46%	7,49%	0,52%	6,91%	0,61%	7,29%	0,68%	N	N	N
H	5,46%	0,34%	5,55%	0,36%	6,30%	0,46%	7,44%	0,62%	7,33%	0,67%	Y	N	N
I	5,67%	0,37%	5,91%	0,38%	6,30%	0,44%	5,79%	0,55%	5,38%	0,59%	N	N	N
J	6,94%	0,41%	6,84%	0,43%	7,63%	0,51%	9,01%	0,66%	9,58%	0,78%	Y	N	N
K	4,83%	0,34%	4,95%	0,35%	4,37%	0,38%	4,82%	0,50%	4,23%	0,51%	N	Y	N
L	8,20%	0,44%	6,00%	0,40%	5,58%	0,44%	5,32%	0,53%	5,75%	0,63%	N	N	Y
M	8,37%	0,43%	8,67%	0,45%	8,42%	0,54%	7,82%	0,65%	8,32%	0,71%	N	N	N
N	3,04%	0,27%	3,04%	0,27%	1,83%	0,25%	1,45%	0,29%	1,36%	0,29%	N	Y	N
O	4,89%	0,33%	4,94%	0,36%	4,67%	0,40%	5,45%	0,55%	5,91%	0,63%	N	N	N
P	6,24%	0,37%	6,40%	0,40%	5,98%	0,46%	5,50%	0,54%	4,58%	0,56%	N	Y	N
Q	3,40%	0,28%	3,50%	0,29%	3,57%	0,35%	2,40%	0,37%	2,32%	0,38%	N	Y	N
R	12,89%	0,56%	13,46%	0,59%	13,37%	0,65%	12,82%	0,82%	11,91%	0,85%	N	Y	N
S	5,38%	0,37%	5,46%	0,36%	5,67%	0,45%	5,16%	0,52%	4,05%	0,50%	N	Y	N
T	4,10%	0,32%	4,11%	0,32%	4,18%	0,38%	4,04%	0,46%	4,05%	0,51%	N	N	N
U	3,25%	0,28%	3,42%	0,29%	3,45%	0,34%	2,89%	0,40%	2,50%	0,41%	N	Y	N
V	1,92%	0,21%	1,91%	0,21%	1,73%	0,24%	1,76%	0,33%	1,14%	0,27%	N	Y	N
