# Supplementary material for: Genomes-based phylogeny of the genus Xanthomonas
Source: BMC Microbiol. 2012 Mar 23;12:43. doi: 10.1186/1471-2180-12-43 (PMC3359215; doi:10.1186/1471-2180-12-43)
Supplement: Additional file 4 — Species counts in similar sequences of cluster 1. Species counts within the BLAST hits in NCBI's NR using the genes of Xeu8 in the cluster as query. [file 1471-2180-12-43-S4.PDF]

156	Pseudomonas aeruginosa
93	Salmonella enterica
48	Bordetella petrii
46	Acidovorax sp. JS42
45	Pseudomonas putida
44	Haemophilus influenzae
43	Pseudomonas fluorescens
40	Pseudomonas syringae group genomosp. 3
34	Xanthomonas campestris
31	Stenotrophomonas sp. SKA14
31	Burkholderia pseudomallei
30	Serratia proteamaculans
30	Pectobacterium wasabiae
28	Aromatoleum aromaticum
27	Escherichia coli
26	Vibrio cholerae
23	Comamonas testosteroni
22	Ralstonia pickettii
22	Proteus mirabilis
21	Xanthomonas axonopodis
21	Pseudomonas amygdali
21	Acidovorax delafieldii
20	Xanthomonas euvesicatoria
20	Thioalkalivibrio sp. HL-EbGR7
20	Methylibium petroleiphilum
20	Congregibacter litoralis
20	Acidovorax citrulli
19	Yersinia pseudotuberculosis
19	Tolumonas auensis
19	Pseudomonas syringae
19	Nitrosomonas eutropha
18	Thauera sp. MZ1T
18	Parvibaculum lavamentivorans
18	Legionella pneumophila
18	Delftia acidovorans
18	Cupriavidus metallidurans
18	Burkholderia xenovorans
18	Acidovorax ebreus
17	Pseudomonas coronafaciens
17	Histophilus somni
16	Nitrosococcus oceani
16	Mannheimia haemolytica
16	Legionella drancourtii
16	Dickeya zeae
14	Pseudomonas savastanoi
13	Erwinia pyrifoliae
13	Edwardsiella ictaluri
12	Pectobacterium atrosepticum
11	Photorhabdus luminescens
11	Klebsiella pneumoniae
11	Citrobacter youngae
11	Azotobacter vinelandii
10	Yersinia enterocolitica
9	Marinobacter hydrocarbonoclasticus
8	Herminiimonas arsenicoxydans
7	Rhodobacter sphaeroides
7	Neisseria gonorrhoeae
6	Haemophilus ducreyi
5	Vibrio splendidus
5	Rhizobium etli
5	Ralstonia solanacearum
5	Legionella longbeachae
4	Xylella fastidiosa
4	Xanthomonas oryzae
4	Vibrio harveyi
4	Shewanella putrefaciens

4	Photobacterium angustum
4	Burkholderia ambifaria
4	Alcanivorax sp. DG881
3	Yersinia intermedia
3	Vibrio vulnificus
3	Verminephrobacter eiseniae
3	Shewanella sp. W3-18-1
3	Shewanella sp. ANA-3
3	Rhizobium leguminosarum
3	Providencia rettgeri
3	Populus trichocarpa
3	Polaromonas sp. JS666
3	Polaromonas naphthalenivorans
3	Photobacterium damselae
3	Hahella chejuensis
3	Citrobacter rodentium
3	Burkholderia multivorans
2	Yersinia ruckeri
2	Vibrio sp. RC341
2	Vibrio sp. Ex25
2	Vibrio mimicus
2	Vibrio fluvialis
2	Thioalkalivibrio sp. K90mix
2	Stenotrophomonas maltophilia
2	Shewanella sp. MR-7
2	Shewanella sp. MR-4
2	Rhodoferax ferrireducens
2	Pseudomonas resinovorans
2	Oceanicola granulosus
2	Janthinobacterium sp. Marseille
2	Idiomarina baltica
2	Gallionella capsiferriformans
2	Dickeya dadantii
2	Dechloromonas aromatica
2	Citrobacter sp. 30_2
2	Citrobacter koseri
2	Burkholderia sp. CCGE1002
2	Burkholderia cenocepacia
2	Allochromatium vinosum
2	Aeromonas hydrophila
1	Zymomonas mobilis
1	Yersinia rohdei
1	Yersinia pestis
1	Yersinia mollaretii
1	Yersinia kristensenii
1	Yersinia frederiksenii
1	Yersinia bercovieri
1	Xanthomonas albilineans
1	Vibrio sp. RC586
1	Vibrio sp. MED222
1	Vibrio sp. AND4
1	Vibrio parahaemolyticus
1	Vibrio metschnikovii
1	Vibrio furnissii
1	Vibrio fischeri
1	Vibrio coralliilyticus
1	Vibrio alginolyticus
1	Variovorax paradoxus
1	Thiomonas intermedia
1	Thiobacillus denitrificans
1	Sinorhizobium medicae
1	Shigella flexneri
1	Shigella boydii
1	Shewanella oneidensis
1	Shewanella baltica
1	Serratia odorifera

1	Serratia marcescens
1	Ruegeria pomeroyi
1	Ruegeria lacuscaerulensis
1	Roseovarius sp. HTCC2601
1	Roseovarius sp. 217
1	Roseobacter litoralis
1	Roseobacter denitrificans
1	Rickettsia typhi
1	Rhodospirillum centenum
1	Rhodopseudomonas palustris
1	Rhizobium sp. NGR234
1	Reinekea blandensis
1	Psychromonas sp. CNPT3
1	Pseudovibrio sp. JE062
1	Pseudomonas stutzeri
1	Pseudomonas sp. TS44
1	Pseudomonas mendocina
1	Pseudomonas entomophila
1	Pseudoalteromonas tunicata
1	Providencia stuartii
1	Providencia alcalifaciens
1	Proteus vulgaris
1	Photobacterium profundum
1	Pectobacterium carotovorum
1	Paracoccus denitrificans
1	Pantoea sp. At-9b
1	Oceanicola batsensis
1	Oceanibulbus indolifex
1	Nitrosospira multiformis
1	Nitrosomonas sp. AL212
1	Nitrosomonas europaea
1	Nitrococcus mobilis
1	Neptuniibacter caesariensis
1	Neisseria meningitidis
1	Moritella sp. PE36
1	Methylovorus sp. SIP3-4
1	Methylophaga thiooxidans
1	Methylobacterium sp. 4-46
1	Methylobacterium populi
1	Mesorhizobium loti
1	Mariprofundus ferrooxydans
1	Marinomonas sp. MWYL1
1	Marinobacter sp. ELB17
1	Marinobacter algicola
1	Magnetospirillum magneticum
1	Magnetospirillum gryphiswaldense
1	Leptothrix cholodnii
1	Kangiella koreensis
1	Idiomarina loihiensis
1	Hoeflea phototrophica
1	Helicobacter pullorum
1	Halorhodospira halophila
1	Gluconobacter oxydans
1	Escherichia sp. 1_1_43
1	Erwinia amylovora
1	Curvibacter putative symbiont of Hydra magnipapillata
1	Cupriavidus taiwanensis
1	Cupriavidus necator
1	Colwellia psychrerythraea
1	Citrobacter freundii
1	Chromohalobacter salexigens
1	Chelativorans sp. BNC1
1	Cellvibrio japonicus
1	Campylobacter jejuni
1	Burkholderia vietnamiensis
1	Burkholderia sp. H160

1	Burkholderia sp. CCGE1001
1	Burkholderia sp. 383
1	Burkholderia graminis
1	Bradyrhizobium japonicum
1	Bermanella marisrubri
1	Beggiatoa sp. PS
1	Azoarcus sp. BH72
1	Arsenophonus nasoniae
1	Alkalilimnicola ehrlichii
1	Alcanivorax borkumensis
1	Agrobacterium tumefaciens
1	Aeromonas salmonicida
1	Acidithiobacillus ferrooxidans
1	Acidithiobacillus caldus
0	unknown
