# Supplementary material for: Genomes-based phylogeny of the genus Xanthomonas
Source: BMC Microbiol. 2012 Mar 23;12:43. doi: 10.1186/1471-2180-12-43 (PMC3359215; doi:10.1186/1471-2180-12-43)
Supplement: Additional file 5 — Species counts in similar sequences of cluster 2. Species counts within the BLAST hits in NCBI's NR using the genes of XamC in the cluster as query. [file 1471-2180-12-43-S5.PDF]

72	Escherichia coli
16	Stenotrophomonas sp. SKA14
14	Salmonella enterica
10	Pseudomonas aeruginosa
7	Magnetococcus sp. MC-1
6	Xylella fastidiosa
5	Xanthomonas phage Xp10
5	Xanthomonas phage Xop411
5	Xanthomonas phage phiL7
5	Photobacterium profundum
5	Comamonas testosteroni
4	Xanthomonas phage Xp15
4	Xanthomonas phage OP1
4	Laribacter hongkongensis
4	Bordetella bronchiseptica
3	Pseudomonas putida
3	Providencia alcalifaciens
2	Shigella boydii
2	Shewanella baltica
2	Ralstonia solanacearum
2	Pseudomonas syringae group genomosp. 3
2	Pseudomonas phage D3112
2	Pseudomonas fluorescens
2	Proteus mirabilis
2	Neisseria meningitidis
2	Magnetospirillum magnetotacticum
2	Magnetospirillum magneticum
2	Klebsiella pneumoniae
2	Delftia acidovorans
2	Acidovorax citrulli
1	Xanthobacter autotrophicus
1	Vibrio cholerae
1	unknown
1	Thioalkalivibrio sp. HL-EbGR7
1	Stenotrophomonas maltophilia
1	Sodalis phage SO-1
1	Shigella sonnei
1	Shigella flexneri
1	Shigella dysenteriae
1	Shewanella sp. W3-18-1
1	Shewanella oneidensis
1	Shewanella frigidimarina
1	Shewanella denitrificans
1	Salmonella phage Fels-1
1	Pseudomonas syringae
1	Pseudomonas phage PAJU2
1	Pseudomonas phage MP29
1	Pseudomonas phage F10
1	Pseudomonas entomophila
1	Providencia rustigianii
1	Populus trichocarpa
1	Polaromonas naphthalenivorans
1	Phage Gifsy-2
1	Oxalobacter formigenes
1	Nitrobacter sp. Nb-311A
1	Neisseria sicca
1	Neisseria flavescens
1	Methylobacterium nodulans
1	Methylobacterium extorquens
1	Methylobacillus flagellatus
1	Magnetospirillum gryphiswaldense
1	Leptothrix cholodnii
1	Klebsiella variicola
1	Hahella chejuensis
1	Escherichia fergusonii
1	Escherichia albertii
1	Enterobacteria phage TLS
1	Enterobacteria phage T1
1	Enterobacteria phage SSL-2009a
1	Enterobacteria phage N15
1	Enterobacteria phage lambda
1	Enterobacteria phage cdtI
1	Desulfovibrio vulgaris
1	Citrobacter sp. 30_2
1	Citrobacter koseri
1	Burkholderia vietnamiensis
1	Burkholderia pseudomallei
1	Burkholderia phage BcepGomr
1	Bradyrhizobium sp. ORS278
1	Bordetella pertussis
1	Bordetella avium
1	Arsenophonus nasoniae
1	Agrobacterium vitis
1	Acidovorax avenae
