# Supplementary material for: Genomes-based phylogeny of the genus Xanthomonas
Source: BMC Microbiol. 2012 Mar 23;12:43. doi: 10.1186/1471-2180-12-43 (PMC3359215; doi:10.1186/1471-2180-12-43)
Supplement: Additional file 6 — Distribution of the BLAST Bit Score (BSR) for several paired comparisons. The genes of Xeu8 were used as reference to build histograms of BSR values here displayed in logarithmic scale (blue). In purple, is the distribution by larger windows of values. In green, is the automatically selected threshold based on the valley of the distribution. Discontinuous purple shows the average threshold, while grey indicates four extreme points of the distribution used to evaluate its topology. [file 1471-2180-12-43-S6.PDF]

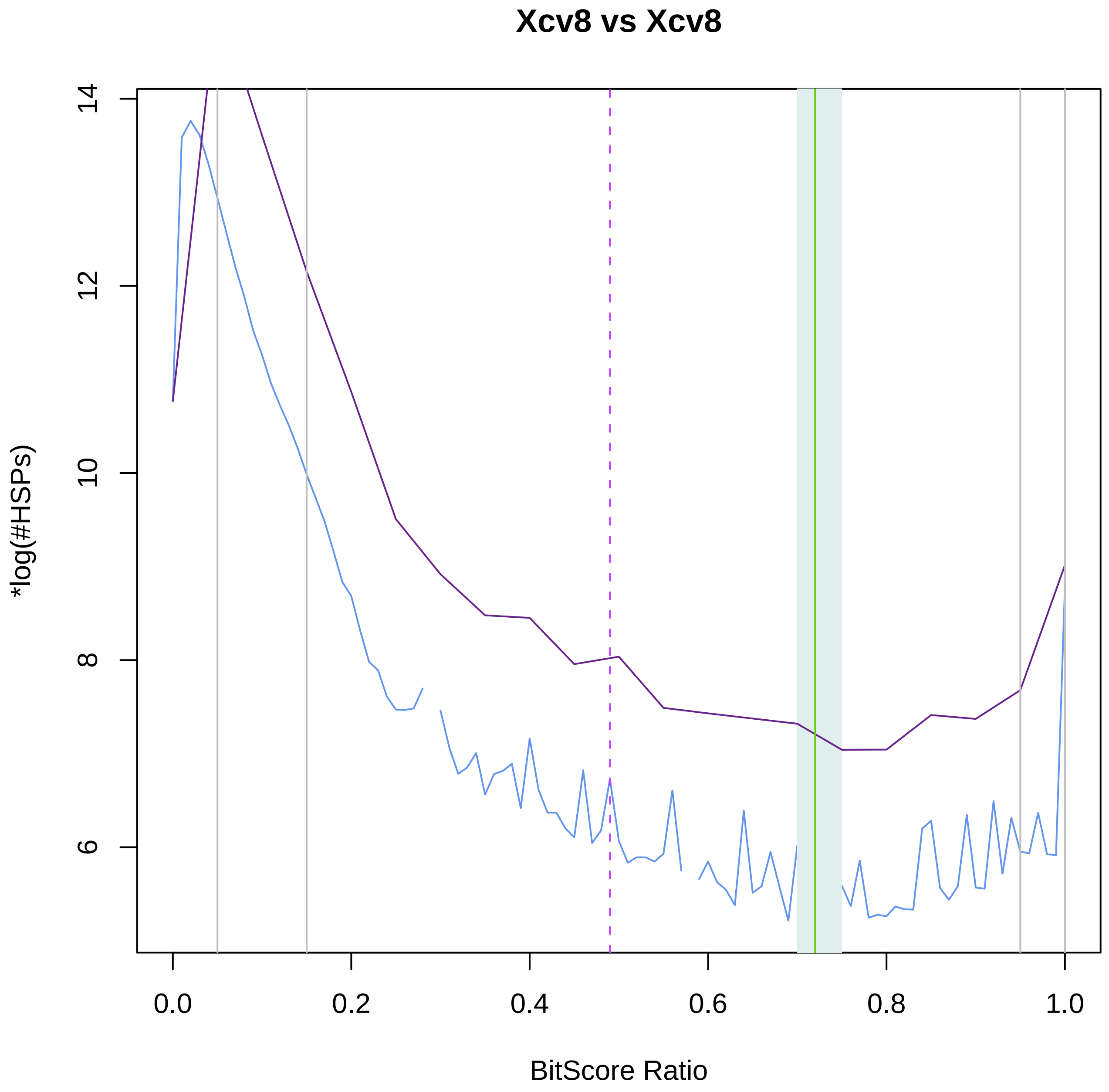
Xcv8 vs Xcv8
*log(#HSPs)
14
12
10
8
6
0.0
0.2
0.4
0.6
0.8
1.0
BitScore Ratio

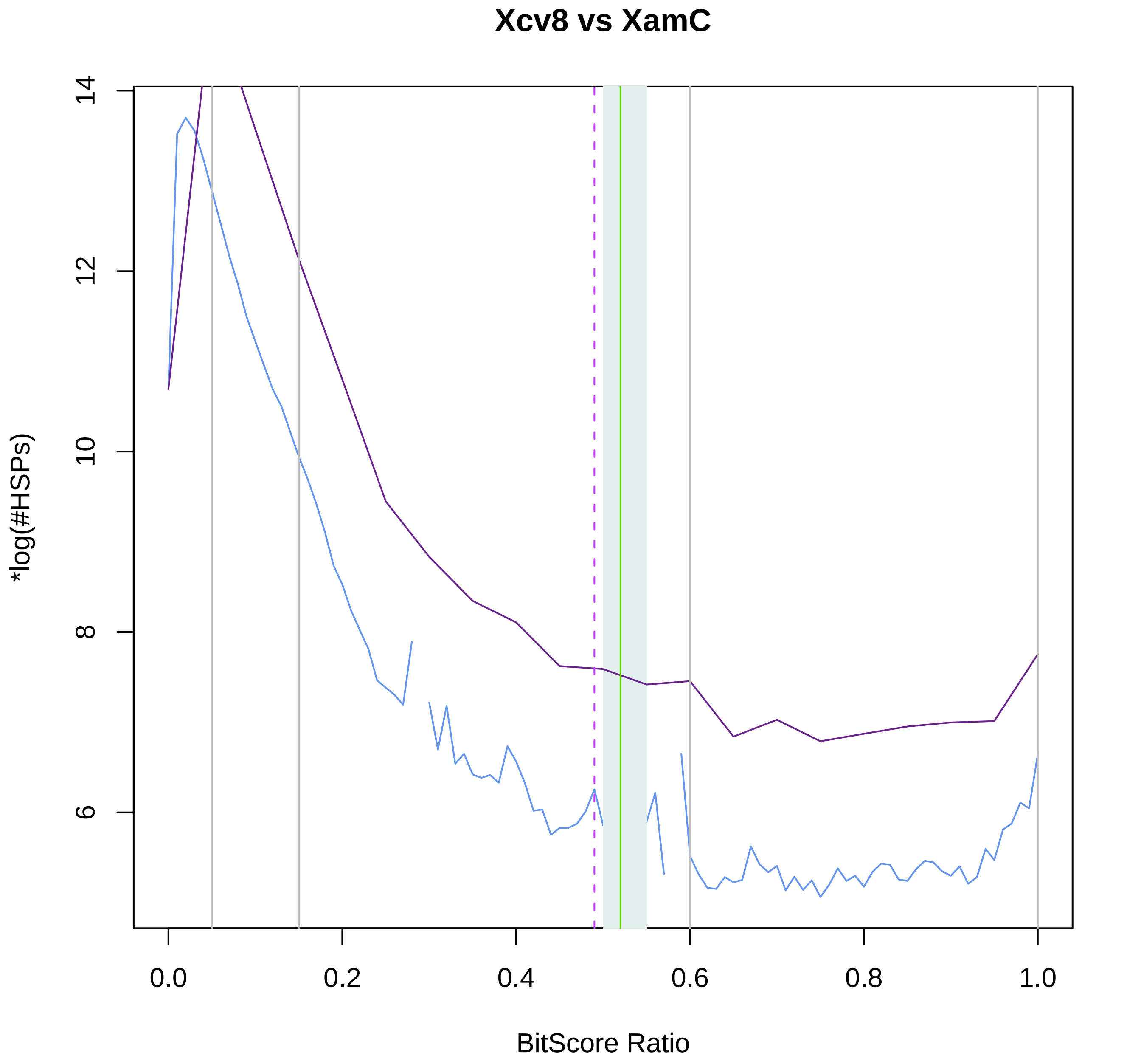

Xcv8 vs XamC
*log(#HSPs)
14
12
10
8
6
0.0
0.2
0.4
0.6
0.8
1.0
BitScore Ratio

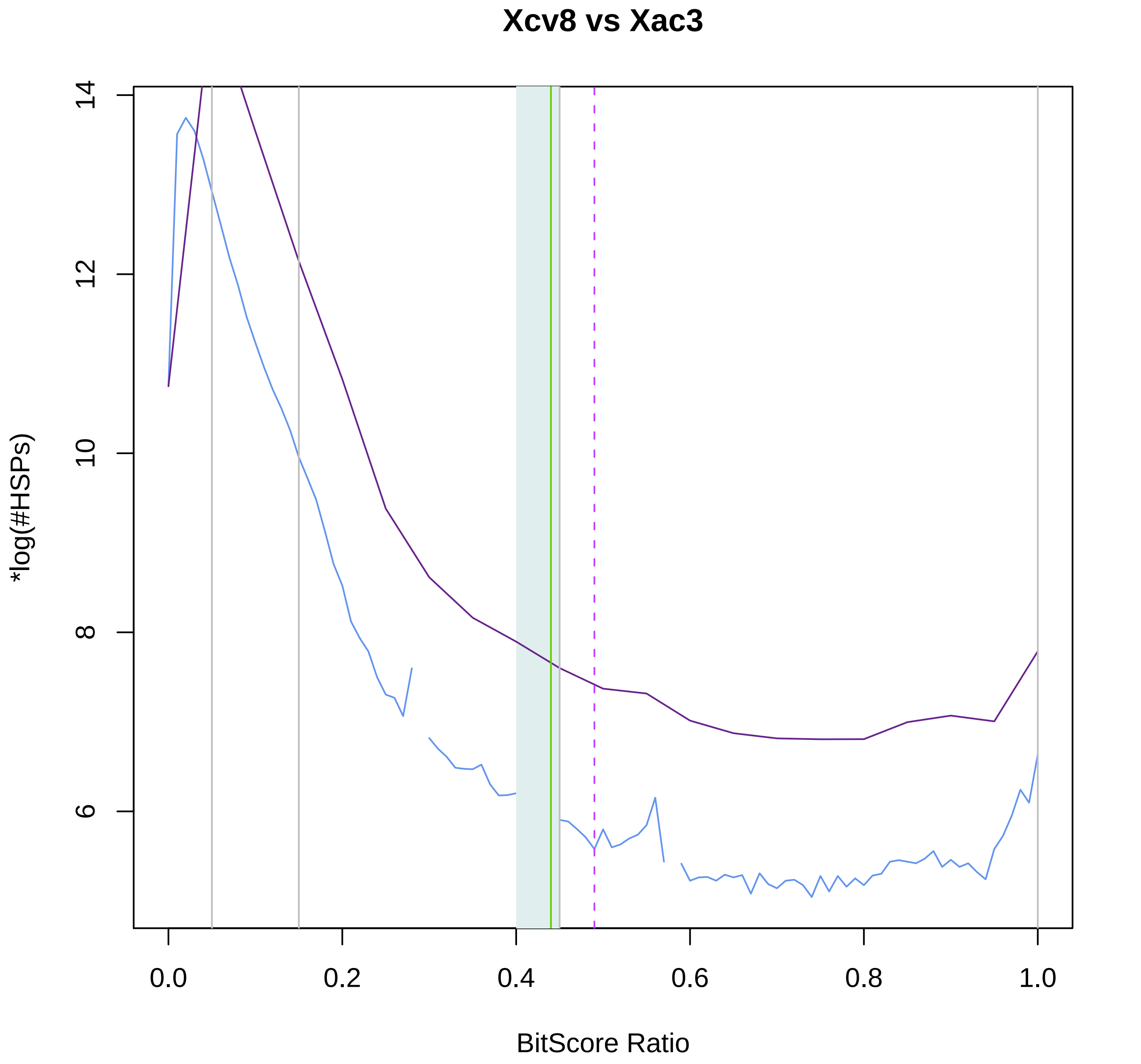

Xcv8 vs Xac3
*log(#HSPs)
14
12
10
8
6
0.0
0.2
0.4
0.6
0.8
1.0
BitScore Ratio

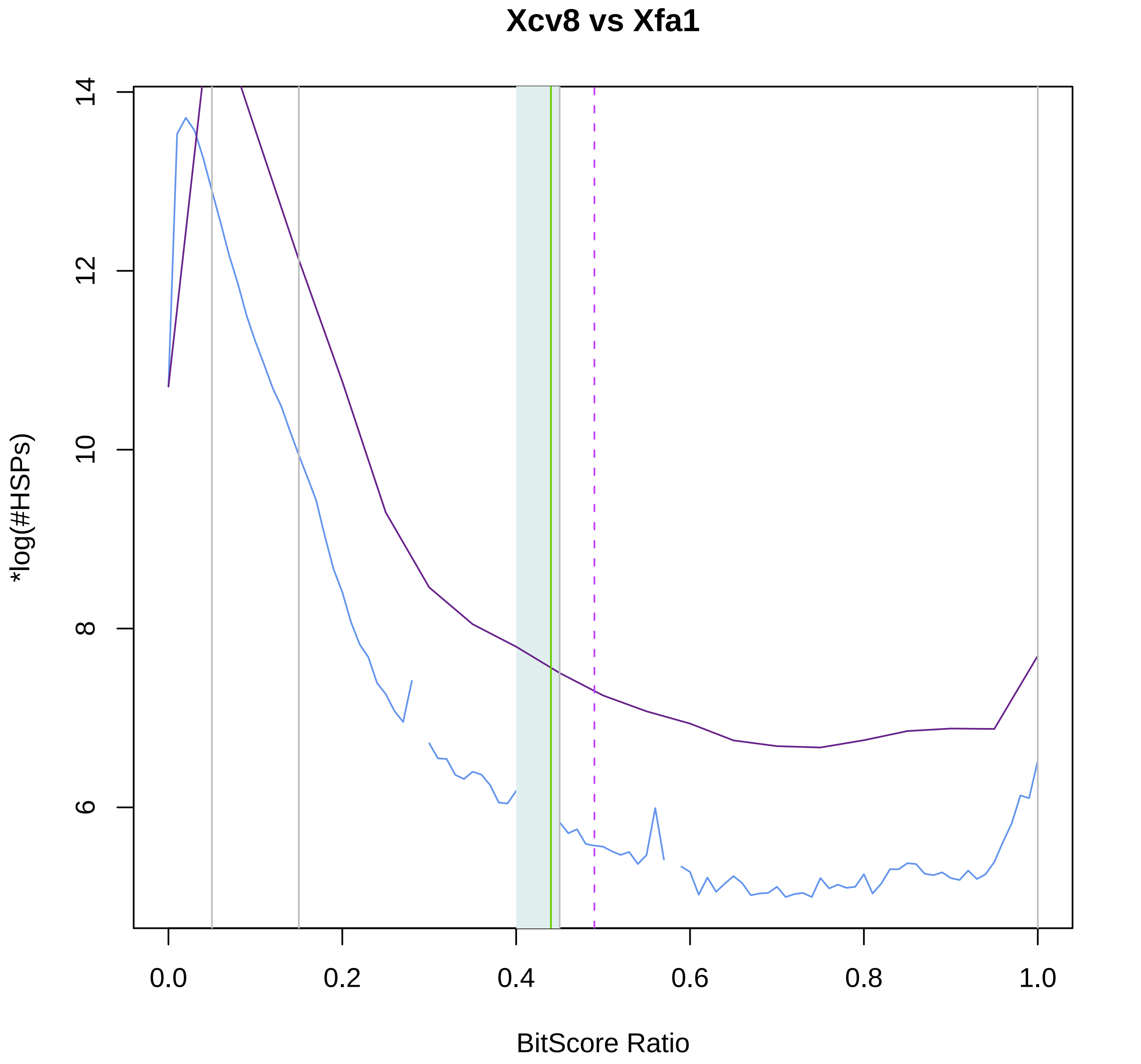

Xcv8 vs Xfa1
*log(#HSPs)
14
12
10
8
6
0.0
0.2
0.4
0.6
0.8
1.0
BitScore Ratio

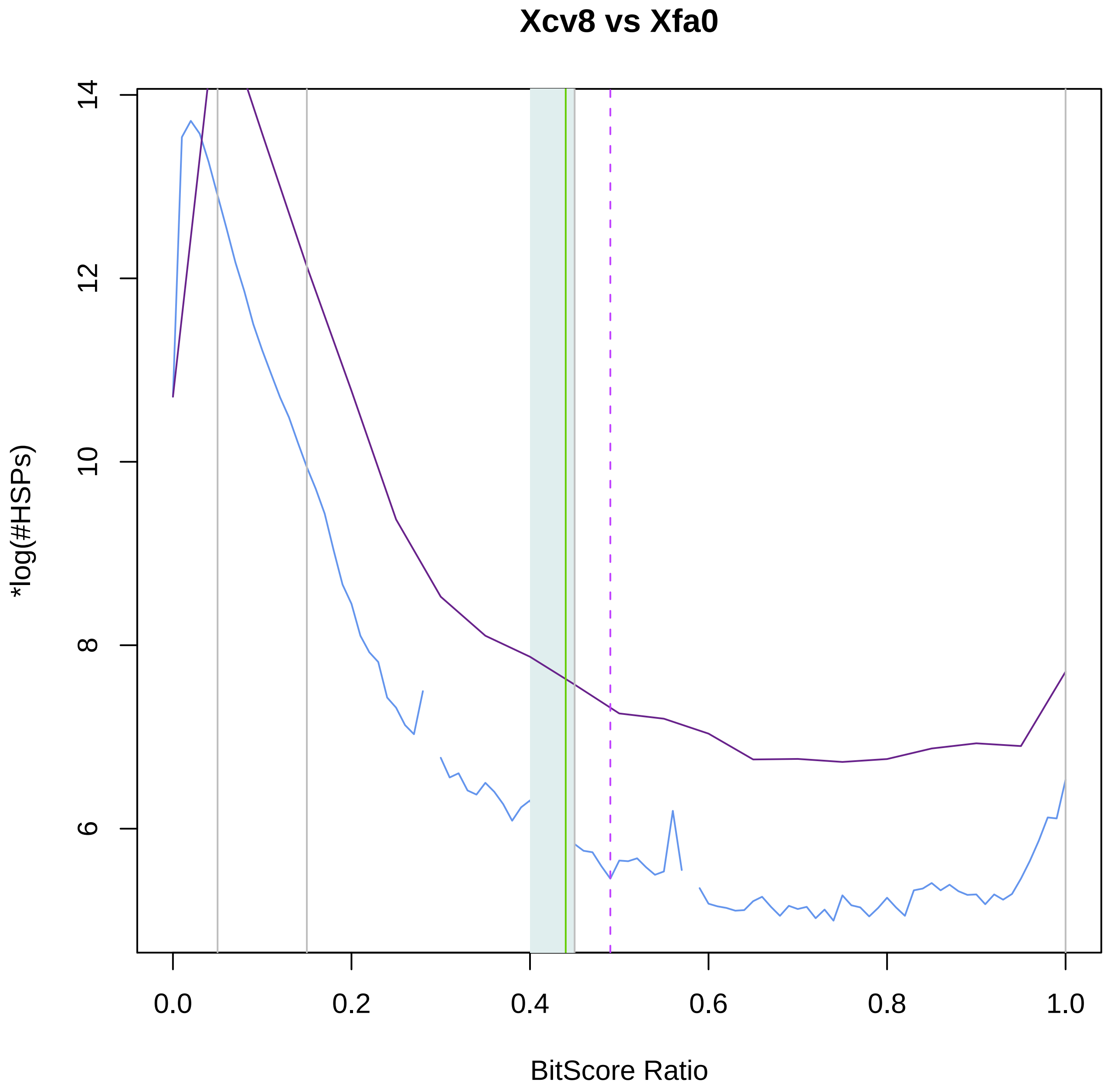

Xcv8 vs Xfa0
*log(#HSPs)
BitScore Ratio
14
12
10
8
6
0.0
0.2
0.4
0.6
0.8
1.0

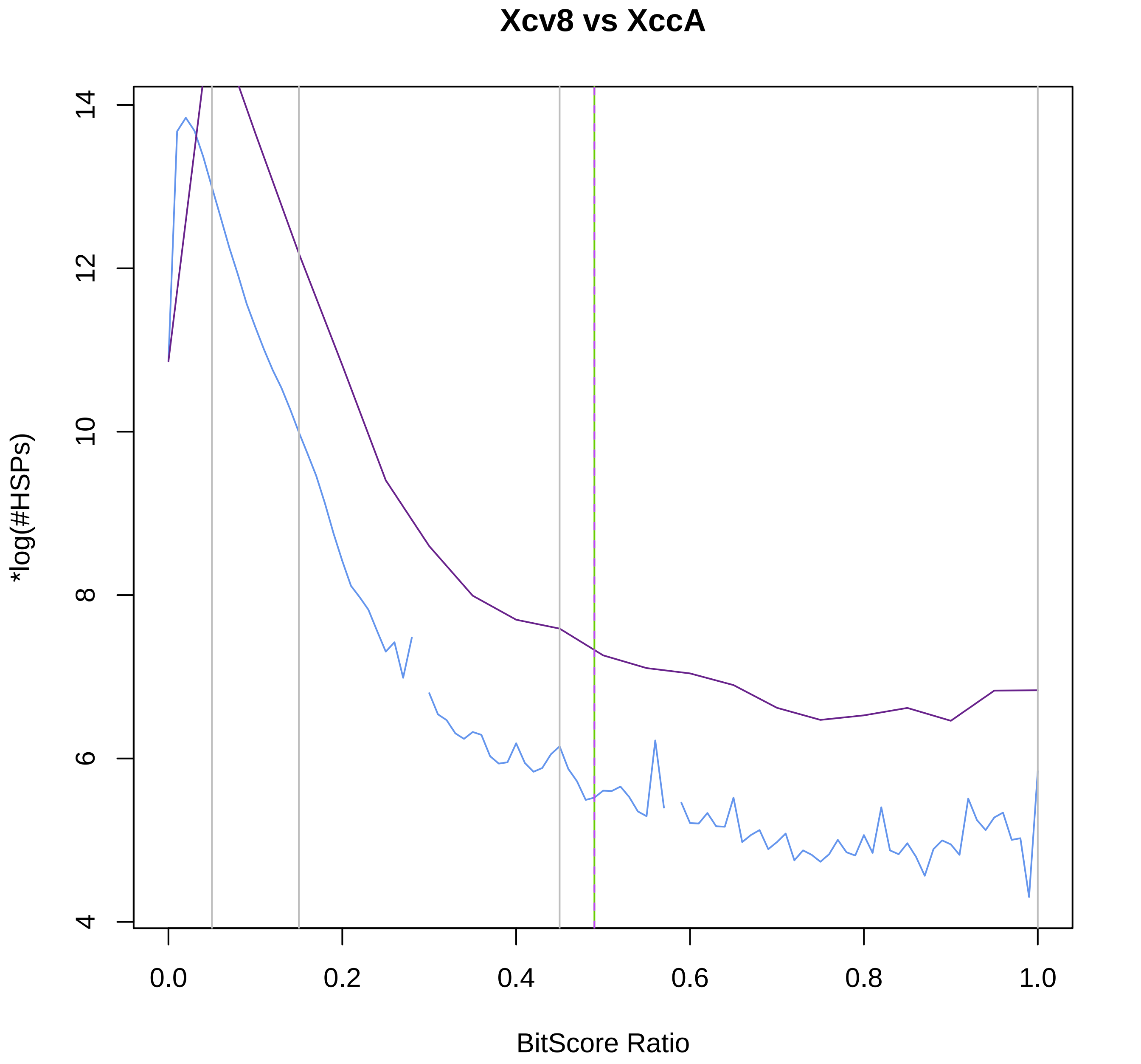
Xcv8 vs XccA
*log(#HSPs)
14
12
10
8
6
4
0.0
0.2
0.4
0.6
0.8
1.0
BitScore Ratio

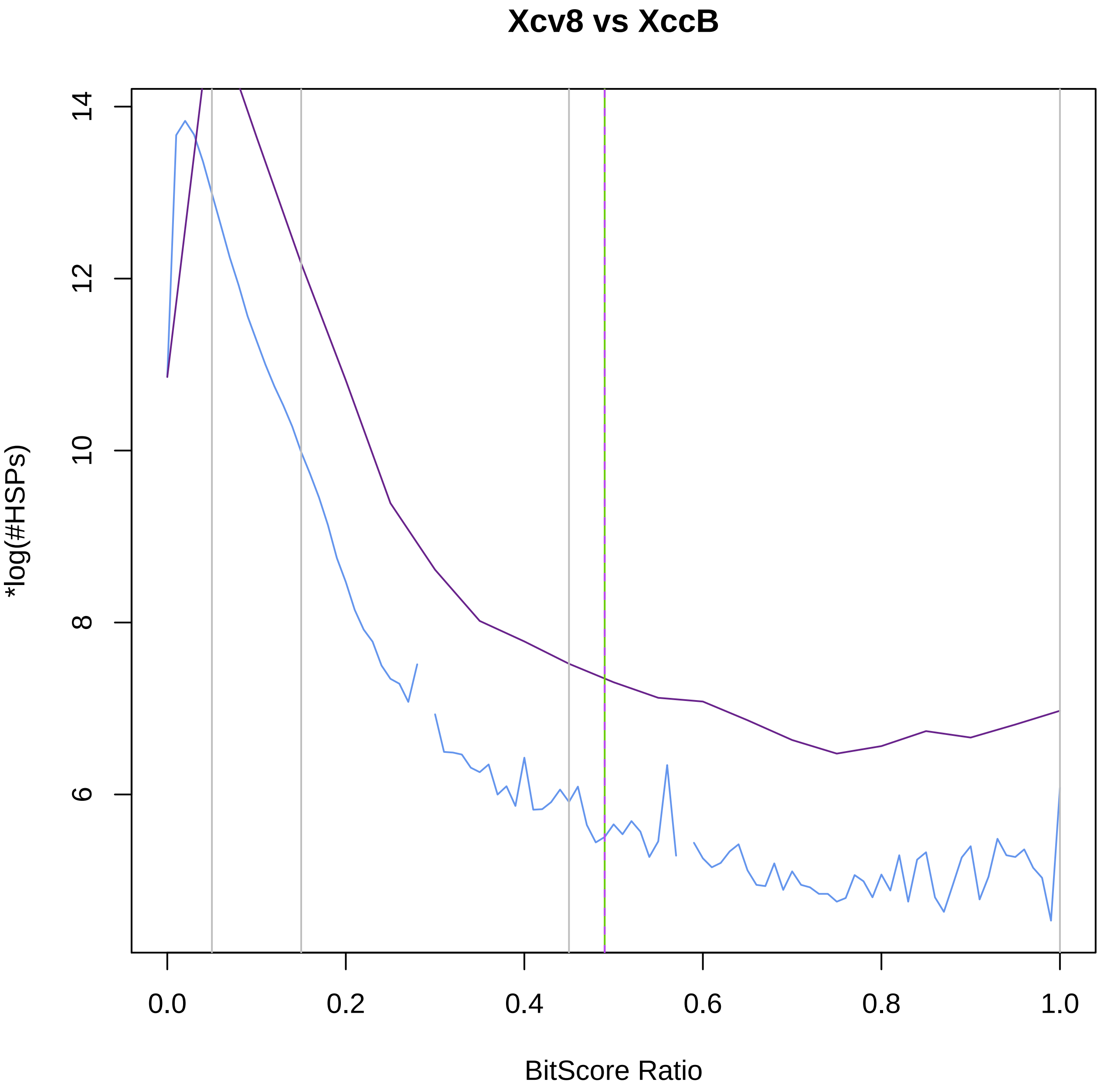
Xcv8 vs XccB
*log(#HSPs)
14
12
10
8
6
0.0
0.2
0.4
0.6
0.8
1.0
BitScore Ratio

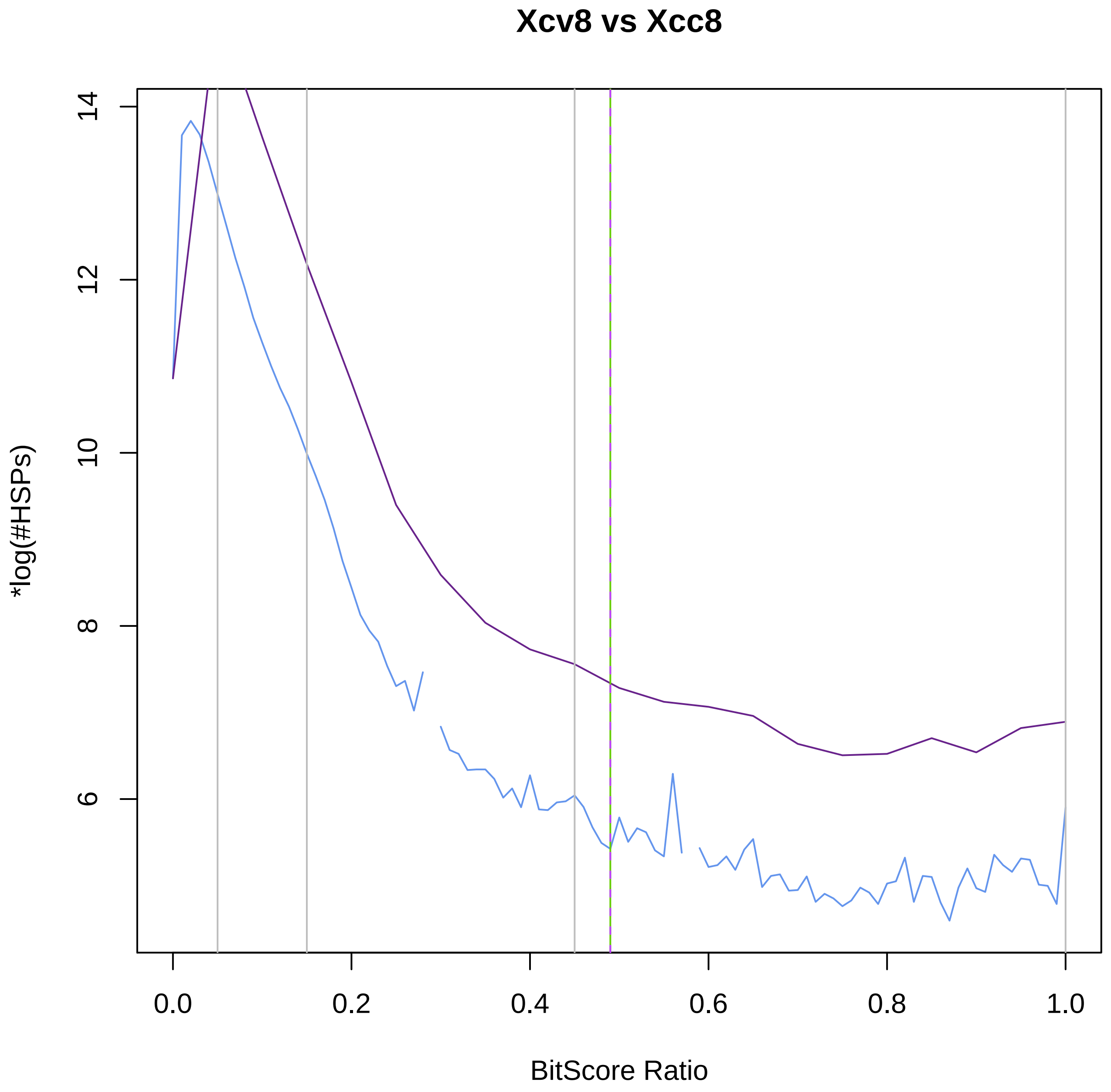

Xcv8 vs Xcc8
*log(#HSPs)
14
12
10
8
6
0.0
0.2
0.4
0.6
0.8
1.0
BitScore Ratio

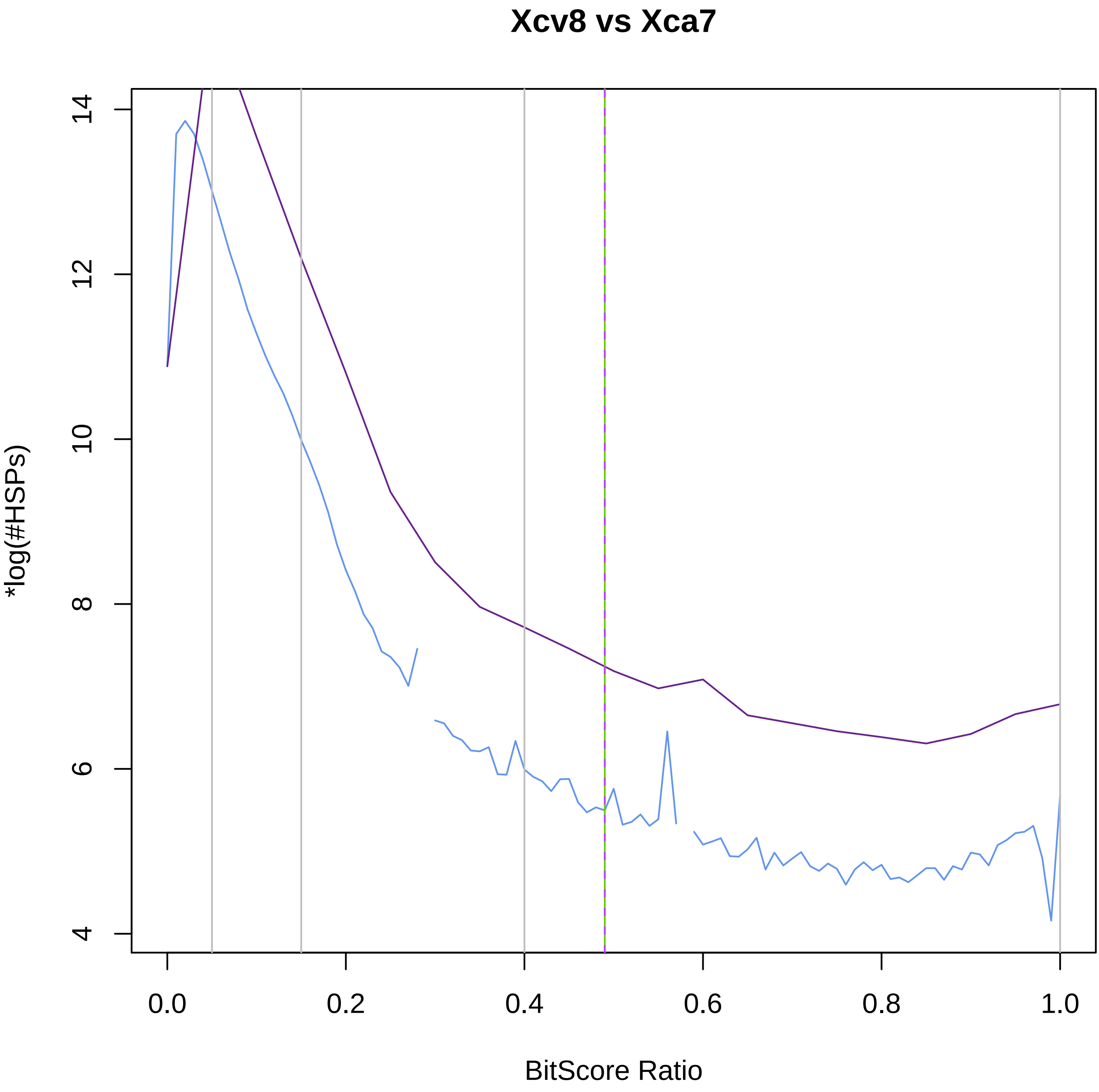

Xcv8 vs Xca7
*log(#HSPs)
BitScore Ratio
14
12
10
8
6
4
0.0
0.2
0.4
0.6
0.8
1.0

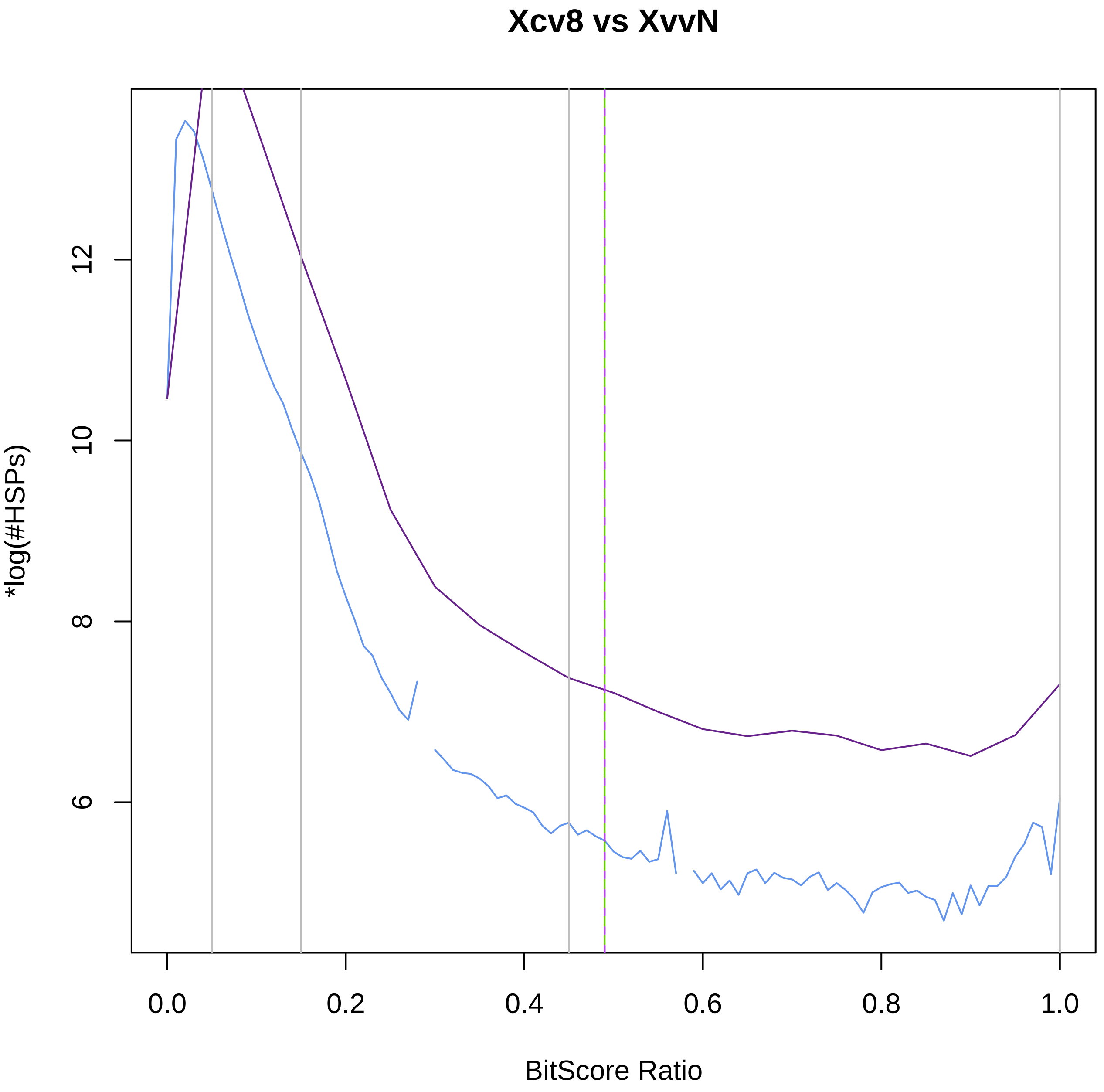

Xcv8 vs XvvN
*log(#HSPs)
6
8
10
12
0.0
0.2
0.4
0.6
0.8
1.0
BitScore Ratio

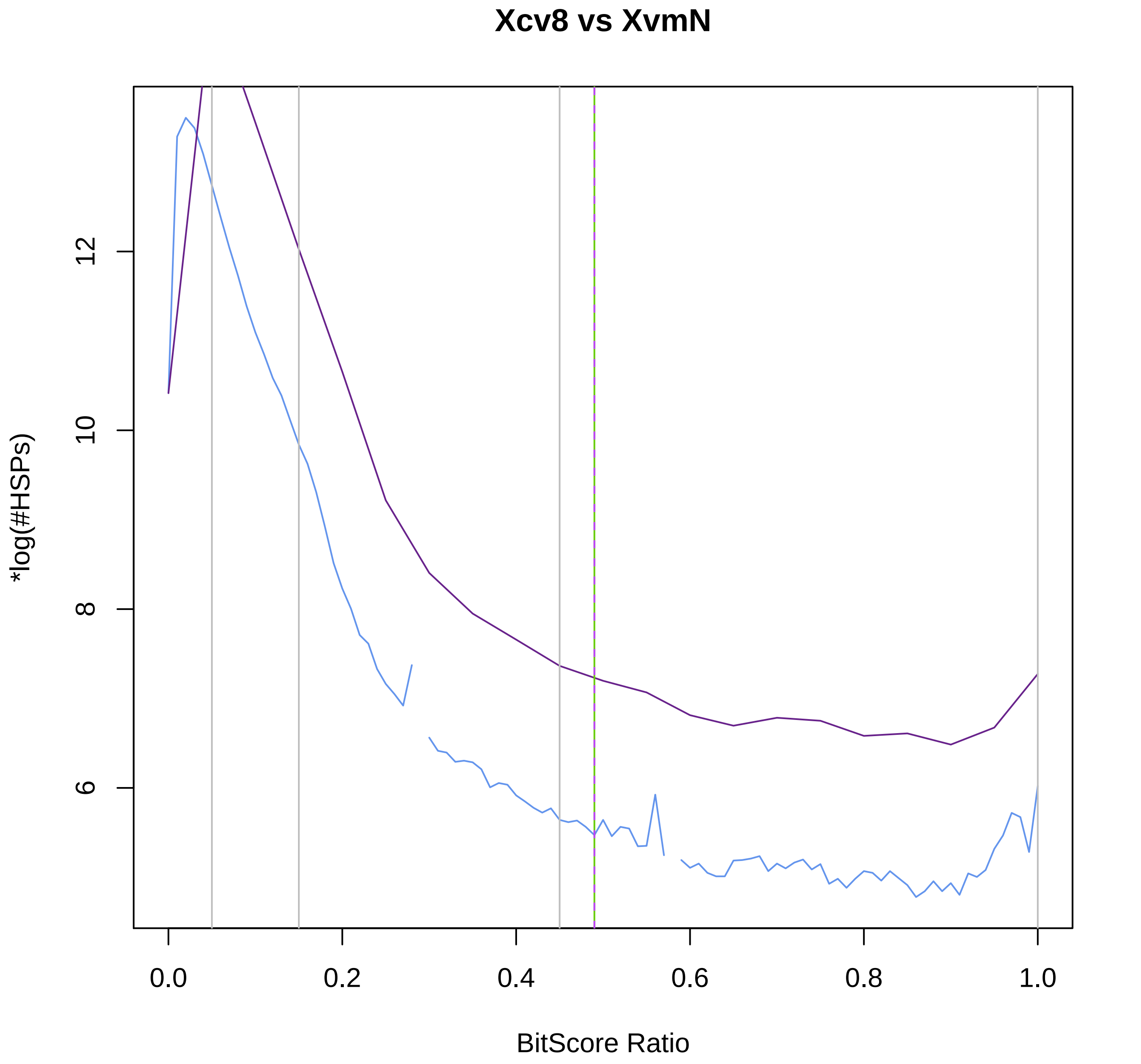

Xcv8 vs XvmN
*log(#HSPs)
6
8
10
12
0.0
0.2
0.4
0.6
0.8
1.0
BitScore Ratio

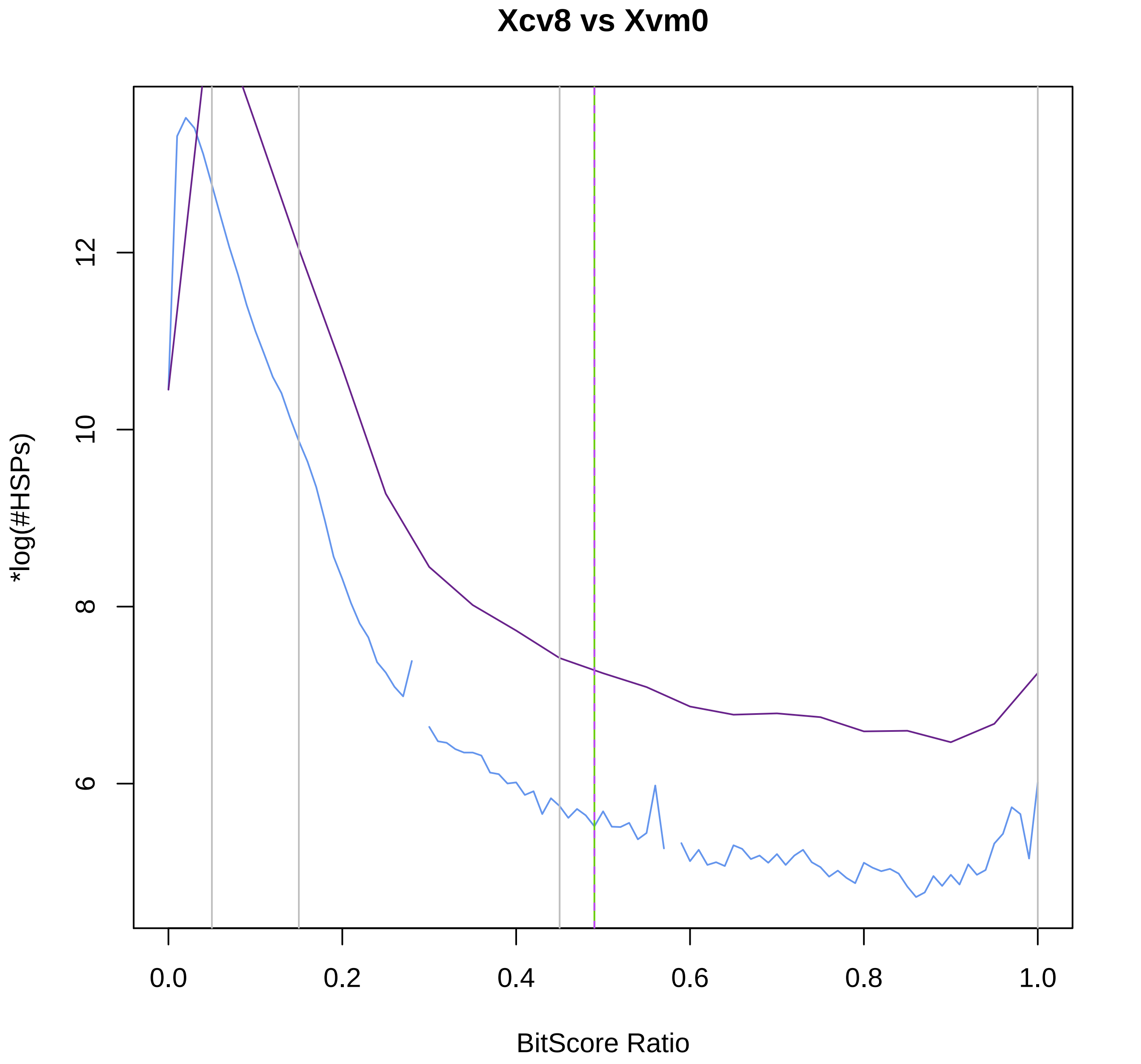

Xcv8 vs Xvm0
*log(#HSPs)
6
8
10
12
0.0
0.2
0.4
0.6
0.8
1.0
BitScore Ratio

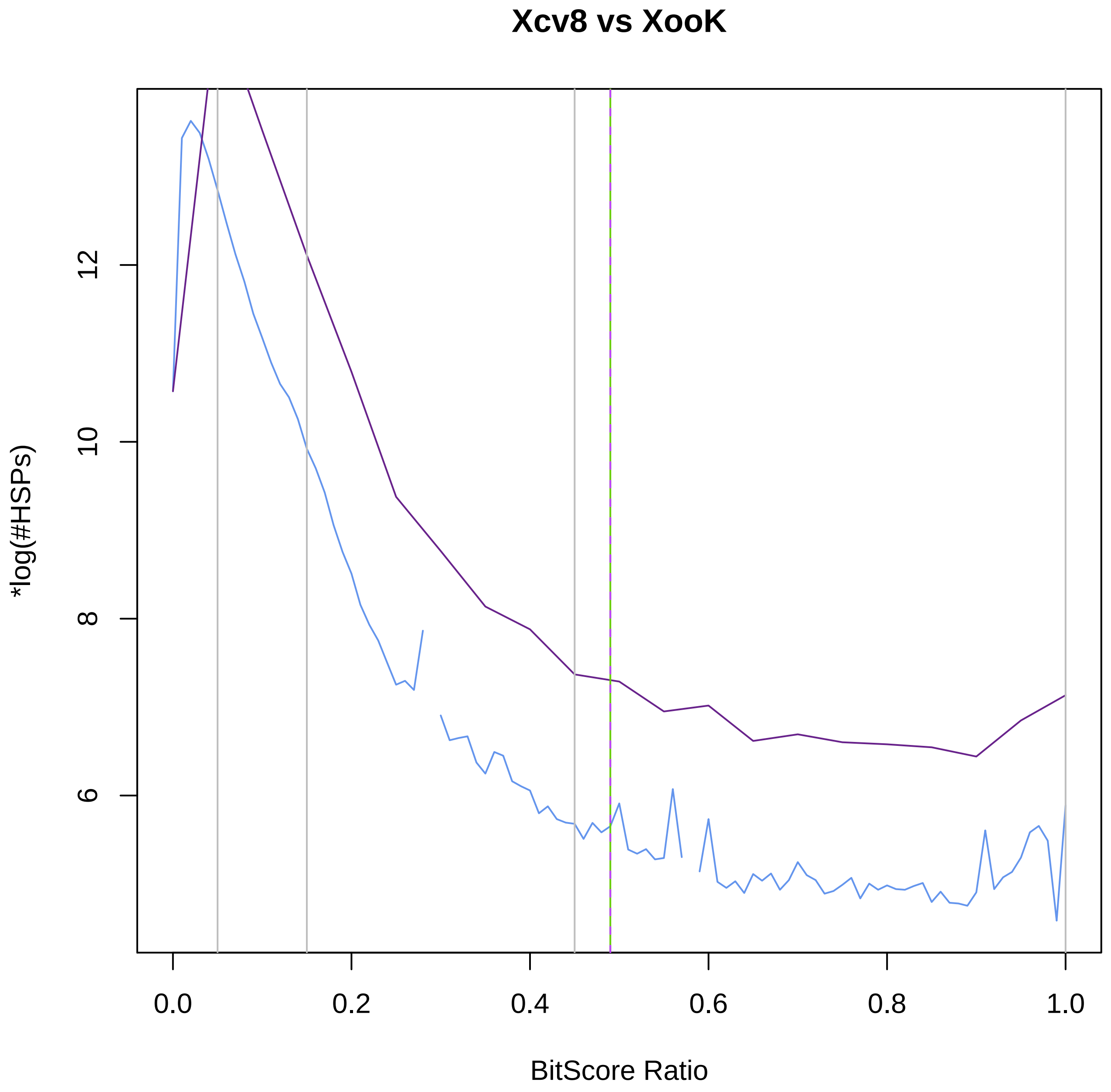
Xcv8 vs XooK
*log(#HSPs)
12
10
8
6
0.0
0.2
0.4
0.6
0.8
1.0
BitScore Ratio

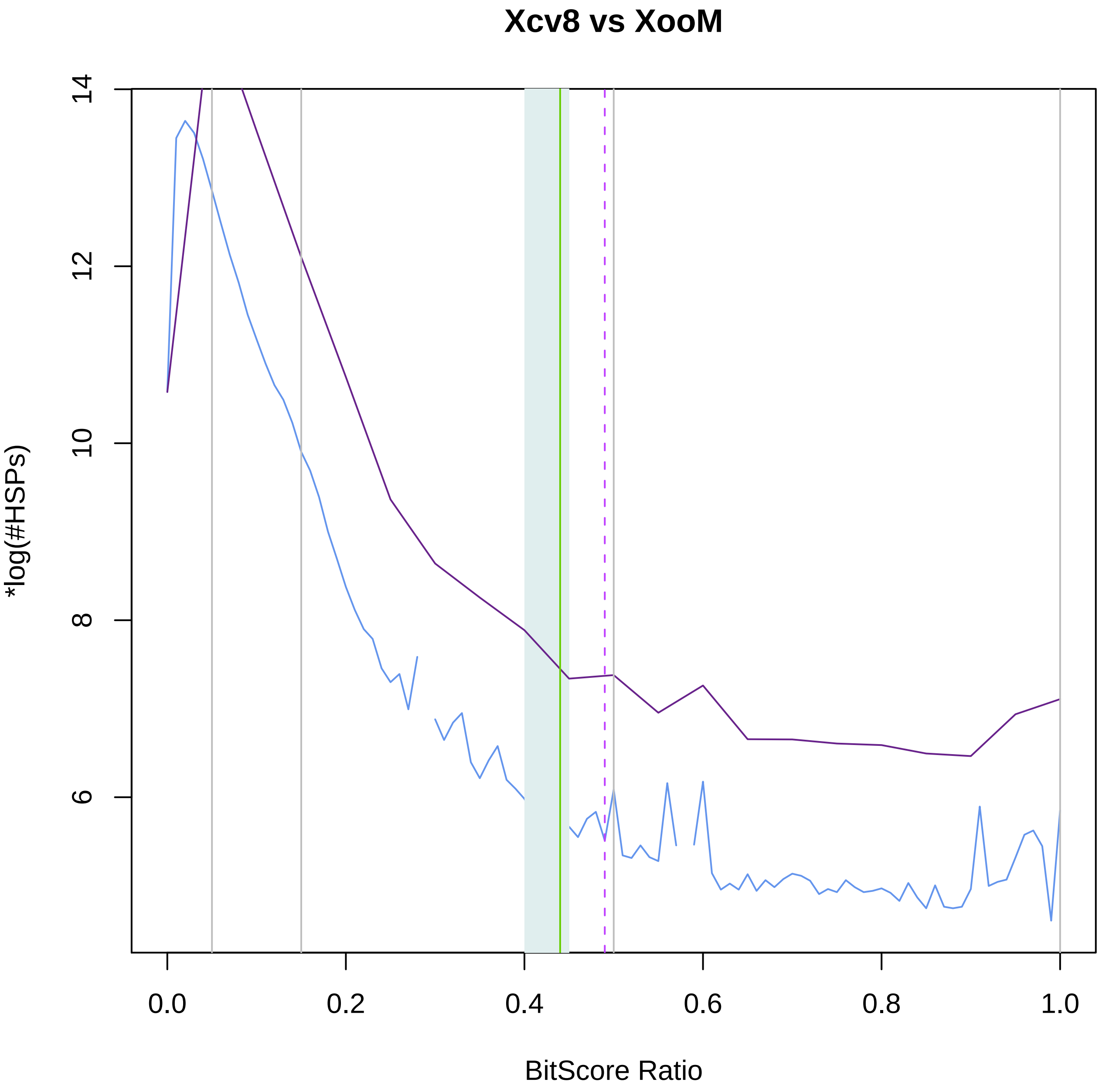
Xcv8 vs XooM
*log(#HSPs)
14
12
10
8
6
0.0
0.2
0.4
0.6
0.8
1.0
BitScore Ratio

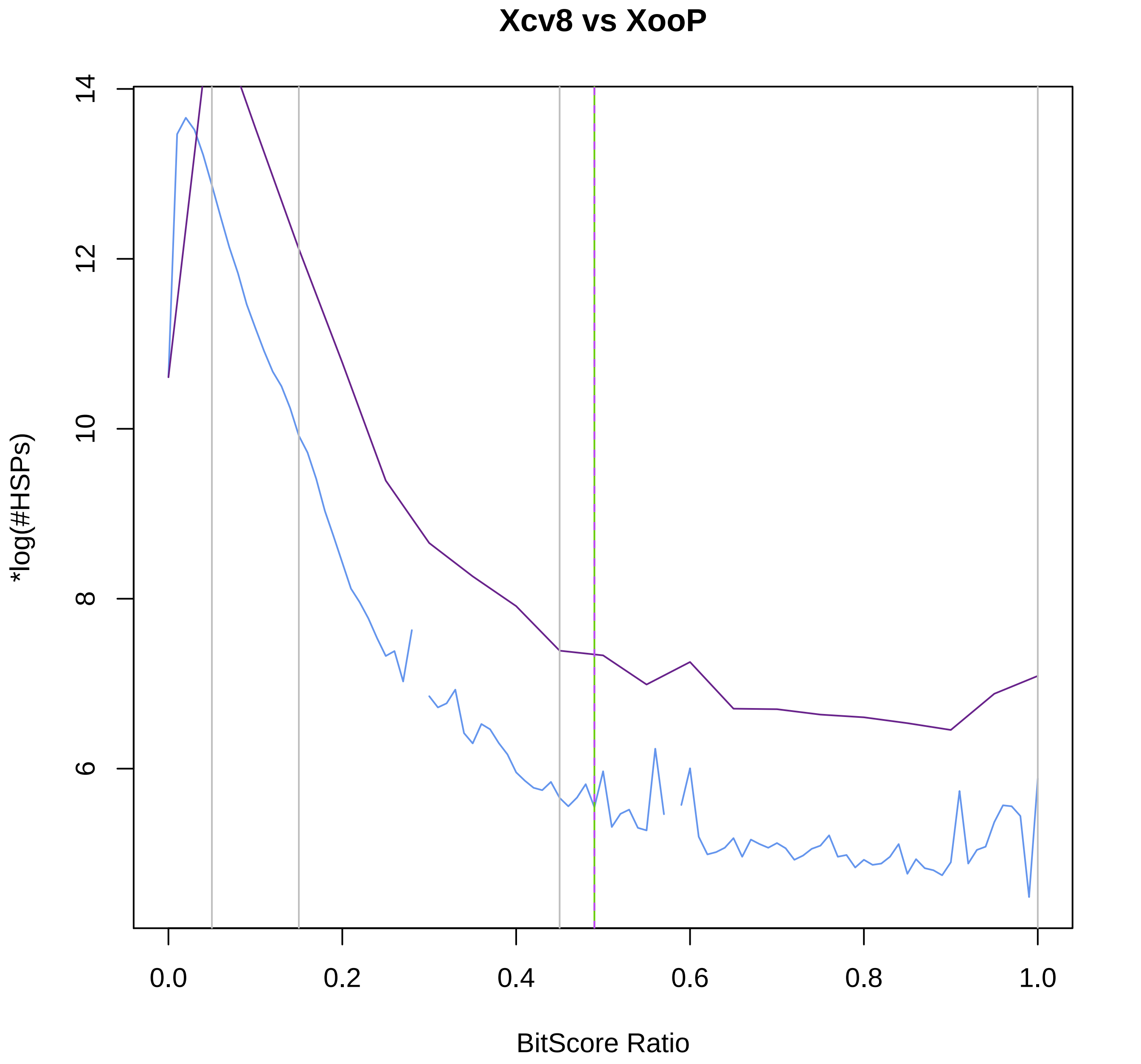

Xcv8 vs XooP
*log(#HSPs)
14
12
10
8
6
0.0
0.2
0.4
0.6
0.8
1.0
BitScore Ratio

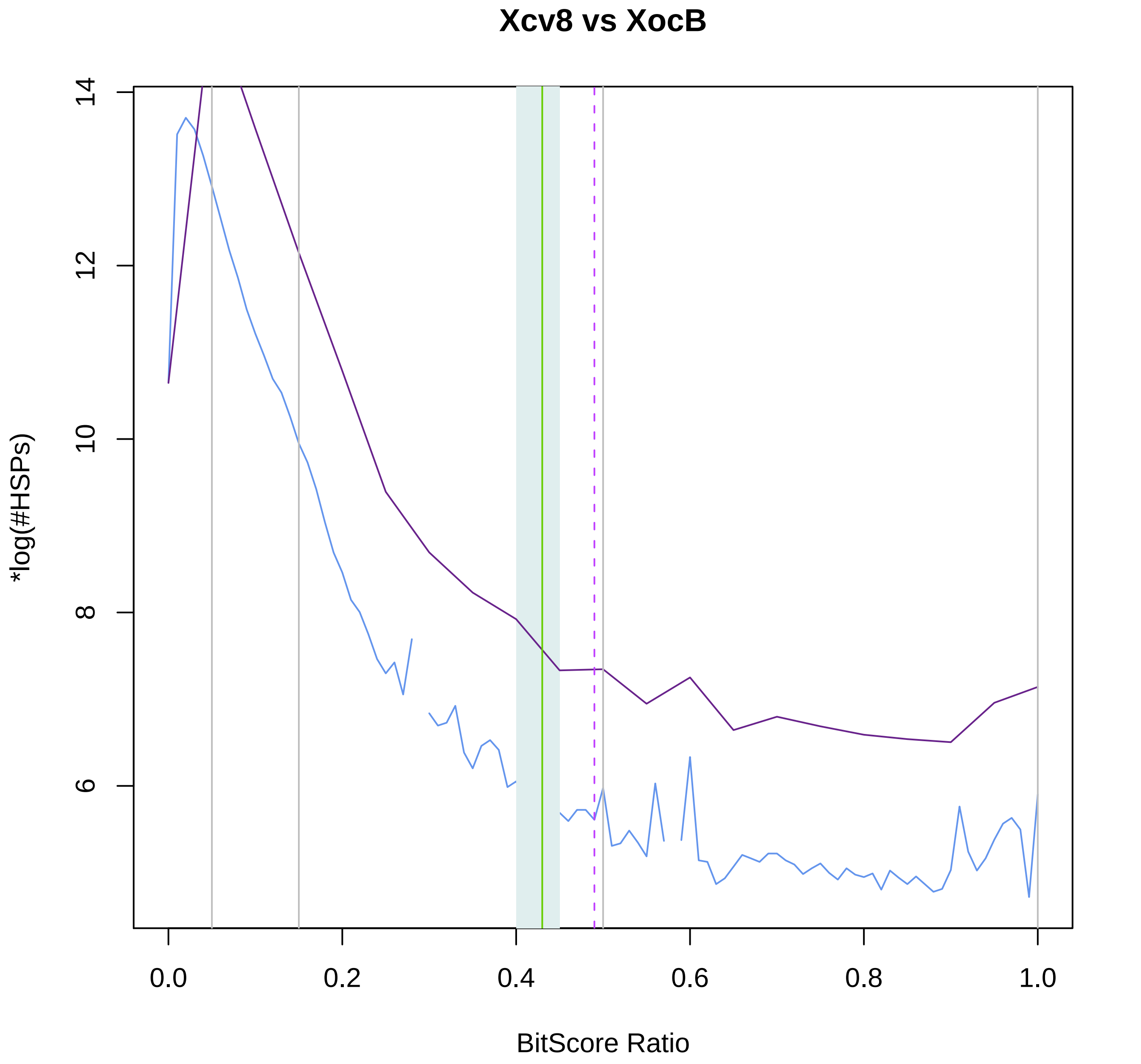
Xcv8 vs XocB
*log(#HSPs)
14
12
10
8
6
0.0
0.2
0.4
0.6
0.8
1.0
BitScore Ratio

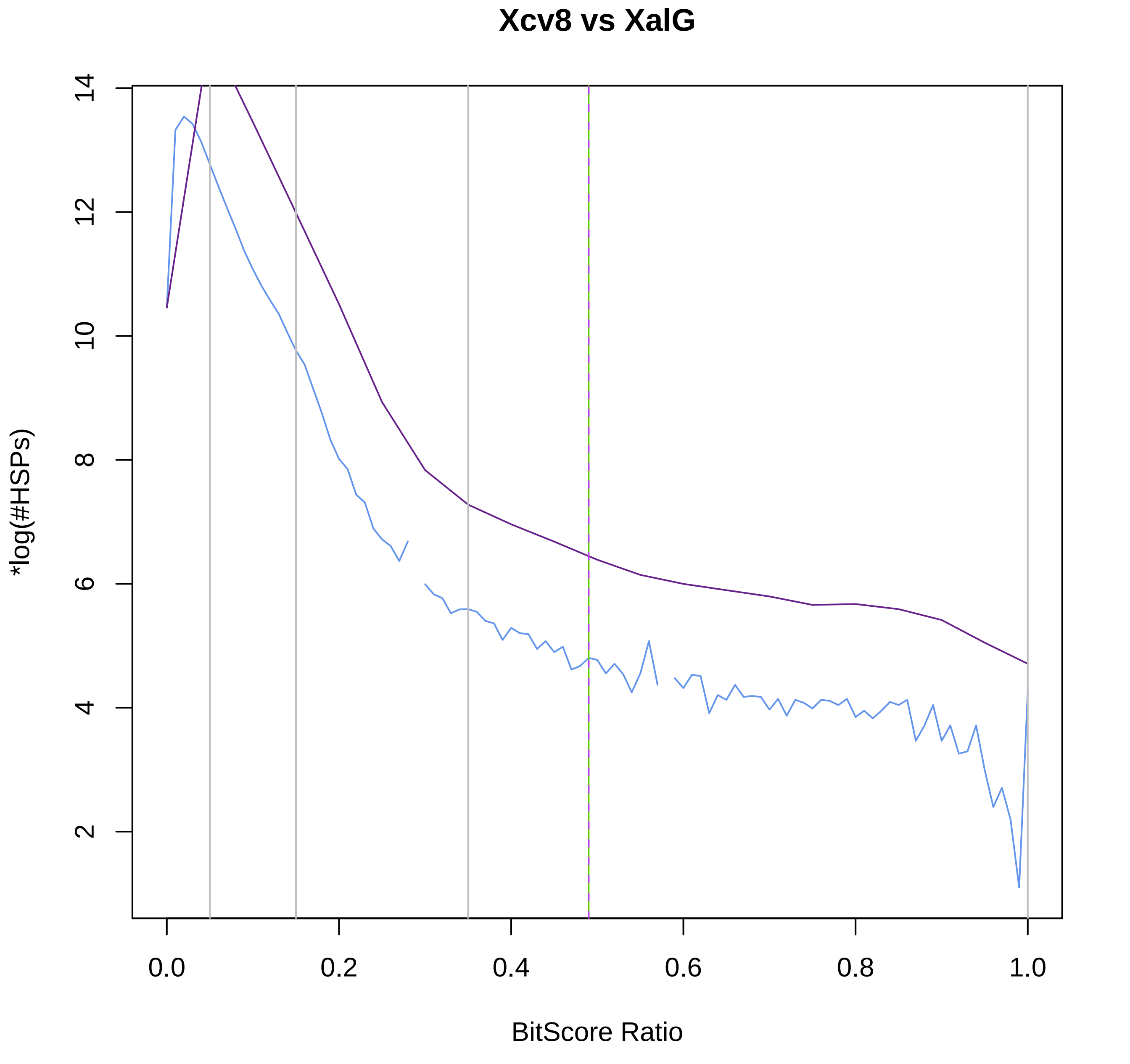

Xcv8 vs XalG
*log(#HSPs)
14
12
10
8
6
4
2
0.0
0.2
0.4
0.6
0.8
1.0
BitScore Ratio
