# Supplementary material for: Genomes-based phylogeny of the genus Xanthomonas
Source: BMC Microbiol. 2012 Mar 23;12:43. doi: 10.1186/1471-2180-12-43 (PMC3359215; doi:10.1186/1471-2180-12-43)
Supplement: Additional file 7 — Supplementary methods. A supplementary text describing methods for the construction of OGs using the Bit Score Ratio with static (BSR-Manual) and dynamic thresholds (BSR-Auto), and the BLAST Reciprocal Best Match (RBM). [file 1471-2180-12-43-S7.PDF]

Supplementary methods

Methods for the construction of OGs

BSR-Manual: BLAST Bit Score Ratio

The BSR is a measure defined as the ratio between the Bit Score of an alignment and the Bit Score of the query sequence aligned with itself (*i.e.*, the highest theoretical bit score achievable by a paired alignment including the query sequence). As previously described [Lerat *et al* 2003; Blom *et al* 2009], this measure has a limited range (zero to one) and a histogram of its values in a paired genomic comparison (using genes as queries) tends to a distribution with two peaks and a valley. The position of that valley has been previously determined in paired comparisons by hand and proposed as a threshold for the acceptance of hits reflecting homology [Lerat *et al* 2003]. They proposed a threshold of 0.3 as a result of their comparisons between genomes of Proteobacteria, with a good trade-off between detection of homologs (sensibility) and exclusion of probably nonspecific matches (specificity). To filter the results using the BSR criterion, we implemented the following procedure in Unus using a BLAST search of the genes in a reference genome (first) against the genes of all the genomes (Ref-vs-all_BLAST):

```
function BSR_OGs(BSR_threshold,Ref-vs-all_BLAST,genomes_list)
      for query ← Each query in Ref-vs-all_BLAST
            Initialize OG with empty vector
            OG ← query
            for hit ← Each hit of query
                  hsp ← Best HSP of hit
                  if hit == query
                        MAX_Score ← score of hsp
                  BSR ← ( score of hsp / MAX_Score )
                  if BSR ≥ BSR_threshold
                        OG ← hit
            if genomes in OG == genomes_list
                  OGs ← OG
      return OGs
```

BSR-Auto: BLAST Bit Score Ration with automatic detection of thresholds

As an attempt to reduce the arbitrary definition of parameters, Unus implements two parallel strategies for the identification of BSR thresholds. The first, aimed to support the manual identification of thresholds, is the production of a script in the R programming language for statistical computing [http://www.r-project.org/] capable of generating a PDF file with the BSR histograms per paired comparison. The second, is an attempt for the automatic detection of thresholds based on such histogram. The implementation in Unus is according to the following pseudo-code:

```
function BSR-Auto_OGs(Ref-vs-all_BLAST,genomes_list)
```

```
        Initialize bsr_hist with empty vector
        Initialize bsr_threshold with empty vector
        for genome_sbjct ← Each genome in genomes_list
            Initialize bsr_hist[genome_sbjct] with empty vector
            for query ← Each query in Ref-vs-all_BLAST
                for hit ← Each hit of query in genomeSbjct
                    bsr_hist[genome_sbjct] ← scores of all the
                                                  HSPs of hit
            if bsr_hist[genome_sbjct] has bimodal distribution
                bsr_threshold[genome_sbjct] ← detect valley in
                                              bsr_hist[genome_sbjct]
        mean_threshold ← Average of bsr_threshold
        for genome_sbjct ← Each genome in genomes_list
            if not bsr_threshold[genome_sbjct]
                bsr_threshold[genome_sbjct] ← mean_threshold
            OG ← BSR_OGs(
                    bsr_threshold[genome_sbjct],
                    hits of Ref-vs-all_BLAST in genome_sbjct,
                    genomes_list)
        return OG
```

In order to optimize resources usage and allow parallelization of the code, several memory variables in the pseudo-code above were replaced by hardcoded files.

RBM: BLAST Reciprocal Best Match

The RBM is a widely used method for the detection of orthologs and the base of several methods for the construction of OGs, including OrthoMCL [Li *et al* 2003]. We implemented a very basic method based on RBM in Unus, using no more than the RBM as orthology criterion and assuming in-paralogs as the only form of paralogy. The definition we used for BRM, however, introduces an extra parameter termed `bit_ratio` in the pseudo-code below, aimed to avoid the exclusion of very similar second matches, possibly causing spurious results. The default value of this term in Unus is 0.95, and the classic RBM definition (*i.e.*, strictly considering the best match only) can be considered a particular case in which this term takes a value of 1.

```
function RBM_OGs(Ref-vs-all_BLAST,genomes_list,min_quality*1,
                                                   bit_ratio)
        for query ← Each query in Ref-vs-all_BLAST
            Initialize OG with empty vector
            OG ← query
            for genome_sbjct ← Each genome in genomes_list
                hsp1 ← Best hsp of query in genome_sbjct
```

```
                hsp2 ← Second best hsp of query in genome_sbjct
                if hsp1 is better than min_quality
                    min_score ← score of hsp1 * bit_ratio
                    if Score of hsp2 ≤ min_score
                        OG ← Hit of hsp1
            if genomes in OG == genomes_list
                OGs ← OG
        return
```
